# Alkaline Stability of LaBO_3_ (B = Co, Ni, Mn, and Fe) Perovskites and Their Application as Bifunctional Oxygen Electrocatalysts for Electrochemical Devices

**DOI:** 10.1021/acs.jpcc.5c03846

**Published:** 2025-08-27

**Authors:** Rambabu Gutru, Daniel Muñoz-Gil, Mohamed Mamlouk, Filipe M. L. Figueiredo

**Affiliations:** † CICECO-Aveiro Institute of Materials, Department of Physics, 5994 University of Aveiro, Aveiro 3810-193, Portugal; ‡ School of Engineering, Newcastle University, Newcastle upon Tyne NE1 7RU, U.K.; § Departamento de Química Inorgánica I, Facultad de Ciencias Químicas, Universidad Complutense, Madrid 28040, Spain

## Abstract

Transition metal perovskites and derived compositions have long been suggested as electrocatalysts for the oxygen electrochemical reactions in alkaline media. This paper presents a study of the alkaline stability of LaBO_3_ perovskites (B = Co, Ni, Mn, and Fe) by exposing powdered samples to a 2M NaOH solution with pH > 14 for variable periods of time. The elemental analysis of the supernatant test solution reveals the presence of Fe only after 48 h reaction time, whereas Co is apparent within the first 12 h. Ni and Mn are detected after 24 h in quantities like those obtained for Co after 12 h. These data suggest that the initial dissolution is primarily determined by the nature of the transition metal, in excellent agreement with calculated Pourbaix equilibrium diagrams. The analysis of the powders by electron microscopy combined with energy-dispersive spectroscopy and X-ray photoelectron spectroscopy confirms the underlying compositional changes, which are particularly important on the surface of the particles, where the transition metal cations tend to be depleted, thereby forming lanthanum-enriched regions. It is concluded from these tests that the chemical stability increases in the series Co < Ni < Mn < Fe. A degradation of kinetic parameters for oxygen reduction and evolution reactions (ORR and OER), assessed by linear scanning voltammetry and time-dependent galvanostatic measurements, is observed with variable magnitude depending on the transition metal, following the same compositional trend of the chemical stability series. The effect of the transition metal on ORR and OER performance is well explained by the e_g_ orbital occupation model. The dissolution of these types of materials in strong alkaline media, potentially aggravated in compositions where lanthanum is partly substituted by alkaline earths, underlies complex compositional changes that may determine the performance and stability of the incorporating device, e.g., fuel cells, metal-air batteries, electrolyzers, or supercapacitors.

## Graphical abstract

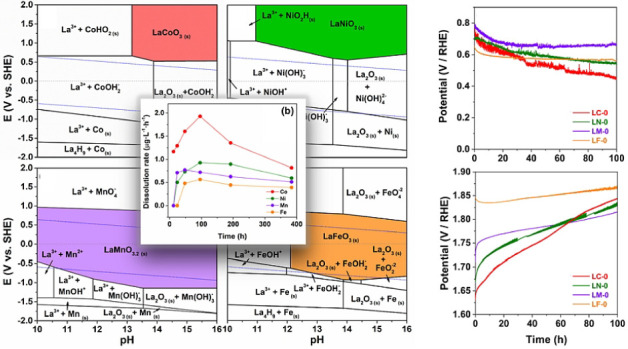

## Introduction

In view of the global energy demand, depletion of fossil fuels, and environmental pollution, there is an urgent need to develop alternative energy devices. In this context, fuel cells, water electrolyzers, and metal-air batteries are key technologies of an alternative, carbon-free energy paradigm based on the electrochemical energy conversion and storage of electricity produced from renewable sources such as solar and wind.^1,2^ The performance of these devices is governed by four fundamental electrochemical reactions, viz., hydrogen evolution reaction (HER), hydrogen oxidation reaction (HOR), oxygen reduction reaction (ORR), and oxygen evolution reaction (OER). The ORR and OER are rate-determining steps in the overall reaction process requiring a large overpotential to follow 4-electron transfer.^3,4^ Platinum (Pt)-based materials are recognized as the most active catalysts for ORR, and they perform poorly toward OER as they form Pt-oxides on the electrode surface under high potential cycling.^5^ Iridium and ruthenium mixed oxides (IrO_2_/RuO_2_) are preferred for OER, but they exhibit poor activity toward ORR.^6^ These materials are scarce and expensive, which are serious drawbacks for massive deployment of the underlying technologies. Electrocatalysis in alkaline media has some advantages over acidic media such as improved reaction kinetics, a less corrosive environment that enhances the durability of the materials and system,^7^ and allows the use of non-noble materials such as transition metal oxides or doped carbons.^8–13^ Perovskite oxides of ABO_3_ type, where A is usually a large lanthanide cation and B a smaller transition metal, have been extensively explored as bifunctional electrocatalysts in alkaline medium due to the abundance of raw materials, economic viability, and the ease of altering the properties by tuning the A site or the B site.^12^ According to the Suntivich et al. model description, the most active perovskite ORR/OER catalyst has an optimal surface oxygen binding energy (i.e., neither too strong nor too weak interactions), and the filling of the e_g_ orbital of the B cation is near unity (e_g_ ≈ 1).^14,15^ However, this cannot be generalized for all the perovskites as most of them are active for OER but not for ORR. For instance, LaMnO_3_ tends to be more active for ORR,^16,17^ while LaCoO_3_ is found to be more active for OER.^18,19^ The reasons may be related not only to oxygen covalency but also to chemical stability. Further to promote the bifunctional activity, partial doping of the B-site and/or the A-site is a promising approach as proved by researchers to enhance the activity of ABO_3_ perovskites. Replacing a small amount of Mn with Co in LaMnO_3_ boosts the OER activity with slight negative impact on ORR,^20^ while A-site doping with Sr improves both ORR and OER activity.^21^ On the other hand, the partial substitution of Co with Fe (typically 5 wt %) expands the potential range of thermodynamic metastability of LaCoO_3_ and stabilizes the Co cations in a lower oxidation state, thus maximizing the available perovskite surface for the formation of an oxy­(hydroxide)­layer under an OER environment.^22^

The long-term (electro)­chemical stability of the electrode materials in an alkaline medium is not addressed in most such studies, being generally assumed. However, this may well not be the case, judging by the few studies available. Ashok et al. studied the stability of LaMnO_3_ and LaCoO_3_ in 1 M KOH under potential cycling and found that the initial current response was reduced to 72% after 2.5 h for both the oxides.^23^ The authors noticed that the current response for cobaltite was stabilized after 2.5 h, while the Manganite shows a decay in response current and hence concluded that, despite the better ORR activity of LaMnO_3_, its long-term stability is poorer than that of LaCoO_3_. Recently, Seok et al. also found evidence to support a degradation mechanism in which the B-site cation species irreversibly leach from LaCo_0.9_Mn_0.1_O_3_, leading to the formation of a 1–2 nm thick amorphous La oxide shell under electrochemical testing conditions in 1 M KOH.^24^

A previous study examined the stability of the perovskite-related Ruddlesden–Popper (RP) phases based on La_2_CuO_4_ and La_2_NiO_4_ in a 2M NaOH alkaline medium.^25^ This study reveals that both Cu and Ni (at a smaller rate) are readily soluble in the alkaline solution even at room temperature and that Sr, a usual substitution for La, dissolves at an even higher rate, in good agreement with calculated Pourbaix diagrams. Kim et al. published in the same year a correlation between the stability and OER activity of Ba_0.5_Sr_0.5_Co_0.8_Fe_0.2_O_3‑δ_ (usually abbreviated as BSCF) and PrBaCo_2_O_5+δ_ (PBCO) using DFT-simulated Pourbaix diagrams.^26^ Although the initial activity of PBCO was higher, it decreased with time due to the dissolution of cations anticipated by the Pourbaix diagrams. In contrast, BSCF shows good structural stability under the OER potentials due to claimed “self-reconstruction” of its surface for the formation of Co-based oxy­(hydroxide) layers. This functional stability was correlated to the thermodynamic metastability of BSCF under the OER conditions suggested by Pourbaix diagrams.

In the present study, we resume the underlying idea of our previous work on the RP phases^25^ to systematically examine the decomposition of LaBO_3−δ_ perovskites (B = Fe, Mn, Co, Ni) in strong alkaline 2M NaOH solutions by analyzing the bulk and surface compositions and to confront these results with calculated Pourbaix diagrams. One further evaluates the ORR and OER activity of fresh and chemically attacked materials to assess the effect of the decomposition on their activity toward oxygen electrocatalysis.

## Experimental Section

### Reagents and Precursors

Lanthanum­(III) nitrate hexahydrate, iron­(III) nitrate nonahydrate, citric acid, and ethylene glycol were purchased from Sigma-Aldrich. Cobalt­(II) nitrate hexahydrate and nickel­(II) nitrate hexahydrate were procured from Merck Chemicals. Manganese­(II) nitrate hexahydrate was procured from Alfa Aesar. Vulcan XC-72R carbon black and Nafion 1100 EW 5 wt % dispersion in alcohol were procured from Ion Power GmbH. A control platinum/carbon (Pt:C) electrocatalyst powder (nominally 40% Pt on carbon black, HiSPEC 4000) was obtained from Johnson Matthey. Sodium hydroxide (ACS reagent >97% purity) pellets were procured from Merck and used for the preparation of 2M NaOH stock solution with deionized water. All chemicals were used as received.

### Synthesis of Perovskite Oxides

LaBO_3±δ_ perovskites with B = Co, Ni, Mn, and Fe, respectively, abbreviated as LC, LN, LM, and LF, were synthesized by a conventional sol–gel method—as described in the literature.^27^ Equimolar ratios of nitrates of La and the transition metal were dissolved in 20 mL of deionized water and then heated to 60 °C under constant stirring. After complete dissolution, citric acid was added to the solution (1.5:1 mass ratio with respect to the metal content) to form the metal complex, followed by the addition of ethylene glycol (1:1 mass ratio) as a stabilizing agent, after which the temperature of the solution is raised to 80 °C until gelation. The obtained gel was dried at 120 °C, thus forming a polyresin which was subsequently fired at 400 °C to remove nitrides and organic residues and at 900 °C to obtain the final product. The resulting powders were grinded in an agate mortar for 30 min and subsequently characterized by powder X-ray diffraction (XRD), nitrogen sorption isotherms, scanning electron microscopy (SEM), transmission electron microscopy (TEM), and X-ray photoelectron spectroscopy (XPS), as detailed elsewhere in this section.

### Calculation of Pourbaix Diagrams

Pourbaix diagrams, plotting the phase stability domains as a function of applied potential and pH of the solution, were calculated following the same procedures reported in ref 25 through the app and the thermodynamic data available in The Materials Project (materialsproject.org, database version Database V2021.11.10). The diagrams are constructed by considering the atomic ratio La:B = 1:1 and variable element concentrations, from 10^–8^ to 10^–2^ mol·kg^–1^. The method involves the combination of experimentally measured free energies of aqueous ions and theoretically determined energies for solid phases and implements corrections for oxygen gas and other liquid phase species and the hydrogen energy.^28,29^ Note that only thermodynamically stable solids are considered for the Pourbaix diagram.

### Chemical Stability Test under Alkaline Conditions

The stability assessment of the perovskite materials under alkaline conditions was carried out by immersion of 1 g of powder of each composition in 20 mL of a 2M NaOH solution (pH > 14) for 12, 24, 48, 96, 192, and 384 h (the number of hours of chemical attack is given as a suffix to the sample acronym for each relevant instance), at room temperature, with a pristine powder sample used for each reaction time test. The suspensions were filtered, and the remnant solutions were analyzed to determine the number of metal cations dissolved. The content of Mn, Ni, and Co was determined by inductively coupled plasma mass spectrometry (ICP-MS, Thermo X Series coupled with autosampler, CETAC ASX510). Due to the high concentration of sodium, the solutions for ICP-MS analysis were diluted 20 times in order to avoid saturation of the detector. The high dilution rate and the overlapping between Fe and Na impose a rather high detection limit for Fe (*ca*. 500 g·L^–1^). The Fe content was thus determined by flame atomic absorption spectroscopy (AAS, GBC Scientific Equipment, model Avanta), which is less prone to the overlap between Na and Fe signals, and thus, the solution could be used with a dilution factor of 4. The blank 2M NaOH solution was also analyzed as a reference. The filtered powders were dried and subsequently analyzed by XRD, SEM, TEM, and XPS to detect the formation of new phases such as hydroxides or changes in the lattice parameters leading to relevant divergences in composition or structure before and after exposure to the NaOH solution. Details of these analyses are given in the following section.

### Structural and Microstructural Characterization of Perovskite Oxides

Powder XRD has been used for identification of the crystalline phase. The data were collected using an Empyrean powder diffractometer with Cu K_α1_ (λ = 1.5406 Å) at room temperature. The patterns were taken in step mode with a step size equal to 0.013 (in 2θ degree unit) and time per step equal to 39 s. The diffraction data were fitted with the Le Bail method using the FullProf software (version July 2017 Copyleft^©^).^30,31^

The morphology of the samples was investigated by SEM (HITACHI SU-70, Hitachi, Ltd. Tokyo, Japan), coupled with energy-dispersive spectroscopy (EDS, Bruker Quantax 400, Bruker AXS Microanalysis, Berlin, Germany). For analysis, the powder was dispersed in methanol at 10 ppm and then suspended on a copper-supported carbon film. For a deeper study of the morphology of the nanoparticles, a Hitachi HD-2700 scanning transmission electron microscope (STEM) equipped with EDS Bruker, operating at an acceleration voltage of 200 kV, was used. For TEM and SEM, the samples were ground in *n*-butyl alcohol and ultrasonically dispersed. A few drops of the resulting suspension were deposited on a carbon-coated grid. The average Feret diameter of the particles (*D*) was determined from the SEM micrographs using ImageJ (v1.52a).

The nitrogen sorption isotherms were obtained using a Micromeritics Tristar 3000 (Micromeritics Instrument Corp., Norcross, GA, USA) porosimeter at 77 K, degassing the samples overnight before the measurements at 423 K and 10 mPa. The specific surface area (*S*_BET_) of the materials was determined through the Brunauer–Emmett–Teller equation.

The surface composition was studied by X-ray photoelectron spectroscopy (XPS) using an ultrahigh vacuum (UHV) system with a base pressure of 2 × 10^–13^ bar. The system is equipped with a hemispherical electron energy analyzer (SPECS Phoibos 150), a delay-line detector, and a monochromatic AlKα (1486.74 eV) X-ray source. High-resolution spectra were recorded at a normal emission takeoff angle and with a pass energy of 20 eV, which provides an overall instrumental peak broadening of 0.5 eV.

### Electrochemical Measurements

The electrochemical properties of the prepared perovskites were tested by a conventional three electrode setup using catalyst inks prepared by mixing the perovskite powder (8 mg) with Vulcan XC-72R (2 mg) carbon black and grounded with a drop of isopropyl alcohol and then transferred to a vail with 1.5 mL of isopropyl alcohol and 0.5 mL of distilled water mixture (3:1 vol. ratio). To this mixture, 40 μL of Nafion 1100 EW 5 wt % dispersion was added as a binder. This mixture was sonicated for 30 min until a homogeneous slurry was obtained. Six μL of this ink was dropped on the surface of a polished glassy carbon rotating disk electrode of 4 mm in diameter (Metrohm Autolab, RDE) and dried at room temperature for 60 min to obtain a thin film with a catalyst loading of 0.2 mg·cm^–2^. Similarly, the ORR control Pt/C was prepared by dropping 5 μL of ink (1 mg·mL^–1^ in deionized water) on the RDE, while the OER control RuO_2_ was prepared by loading 0.2 mg·cm^–2^ of the pure oxide. Linear sweep voltammetry (LSV) was carried out at room temperature using a Metrohm Autolab potentiostat model 302N. The voltammograms were recorded in 0.5 M aqueous KOH electrolyte saturated with oxygen, using a Pt wire as the counter electrode, and Ag/AgCl with 3.5 M KCl as the reference electrode. ORR activity was measured between the potential 0.3 and 1 V vs. RHE at a scan rate of 5 mV·s^–1^ under variable rotation rates from 400 to 2000 rpm, while OER activity was measured between 1.2 and 2 V vs. RHE at a scan rate of 5 mV·s^–1^. The ORR LSVs were analyzed in light of the Koutechý–Levich model by plotting the inverse of the current density *j* ^–1^ (*j* in mA·cm^–2^) as a function of the inverse square route angular velocity ω^–1/2^ (ω in rad·s^–1^), according to the underlying relation

1 $$j^{-1}={\left(nFkC_{O_{2}}\right)}^{-1}+{\left(0.62nFC_{O_{2}}D_{O_{2}}^{2/3}\mu^{-1/6}\right)}^{-1}\cdot \omega^{-1/2}$$

where *n* and *k* are the number of electrons and the rate constant of the ORR, respectively, *F* is the Faraday constant (96485 C·mol^–1^), $C_{O_{2}}$ is the saturation concentration of O_2_ in 0.5 M KOH at 1 atm O_2_ pressure (1.03 × 10^–6^ mol·cm^–3^),^32^ $D_{O_{2}}$ is the diffusion coefficient of O_2_ (1.6 × 10^–5^·cm^2^ s^–1^),^33^ and μ is the kinematic viscosity taken as the ratio between the dynamic viscosity and the density of the 0.5 M KOH solution (9.00 × 10^–3^ cm^2^·s^–1^).^34^

## Results and Discussion

### Composition, Structure, Microstructure, and Stability in Alkaline Media

XRD patterns collected at room temperature reveal a single-phase and their profiles are consistent with perovskite structures of different symmetries depending on the cation in the B position (Figure S1). The patterns corresponding to B = Co, Ni, and Mn can be indexed using a rhombohedral system with space group R*-3c*, as previously reported.^35^ The Fe perovskite exhibits an orthorhombic space group of P*bnm*, also in agreement with previous work.^36^ The refined lattice parameters are given in Table S1.

The treatment in 2M NaOH solution has no noticeable effect on the XRD patterns of the powders, which display solely the underlying perovskite reflections (Figure S1). Moreover, the differences in the lattice parameters between the pristine samples and those attacked by the alkaline media may be considered negligible within the natural dispersion of the data (Table S1). Such kind of apparent “no-effect” of the alkaline treatment was also reported in our previous work on the closely related Ruddlesden-Popper phases.^25^

However, the elemental analysis of the supernatant solution by ICP-MS reveals a significant amount of B-site transition metal (Figure 1a), indicating that the four materials end up dissolving in the alkaline solution. If lower concentration means higher stability, the figure depicts a trend for stability in alkaline media, which decreases in the series Fe > Mn > Ni > Co. One could detect Fe only after 48 h reaction time, whereas the presence of Co is apparent within the first 12 h. Ni and Mn appear after 24 h in quantities like those obtained for Co after 12 h. These data thus suggest that the initial dissolution (up to about 100 h) is primarily determined by the nature of the transition metal. Interestingly, the time dependence of the concentration of the four cations in NaOH solutions follows approximately parallel lines after 96 h, indicating similar dissolution rates after the initial variability. This is perhaps better revealed by the plot of the concentration of the cations normalized to the reaction time, which expresses a dissolution rate (Figure 1b). While the trend is similar for the four materials, the case of Co stands out as it presents a significantly higher and wider range of dissolution rate values, reaching a maximum of 1.93 μg·L^–1^·h^–1^ at a reaction time of 96 h, then decreasing to 0.82 μg·L^–1^·h^–1^ at 384 h. The Ni perovskite has the second-highest dissolution rate (0.93 μg·L^–1^·h^–1^ also at 96 h), followed by Mn (0.77 μg·L^–1^·h^–1^ at 48 h) and Fe (0.77 μg·L^–1^·h^–1^ again at 96 h). At 384 h reaction time, which is the limit of the test duration, the relative order of dissolution is maintained, decreasing according to Co > Ni > Mn > Fe. However, the time dependence of such a decrease also tends to decrease in the same compositional order, which might indicate the onset of a common limiting factor, e.g., the formation of a passivating surface layer.

**Figure 1:**
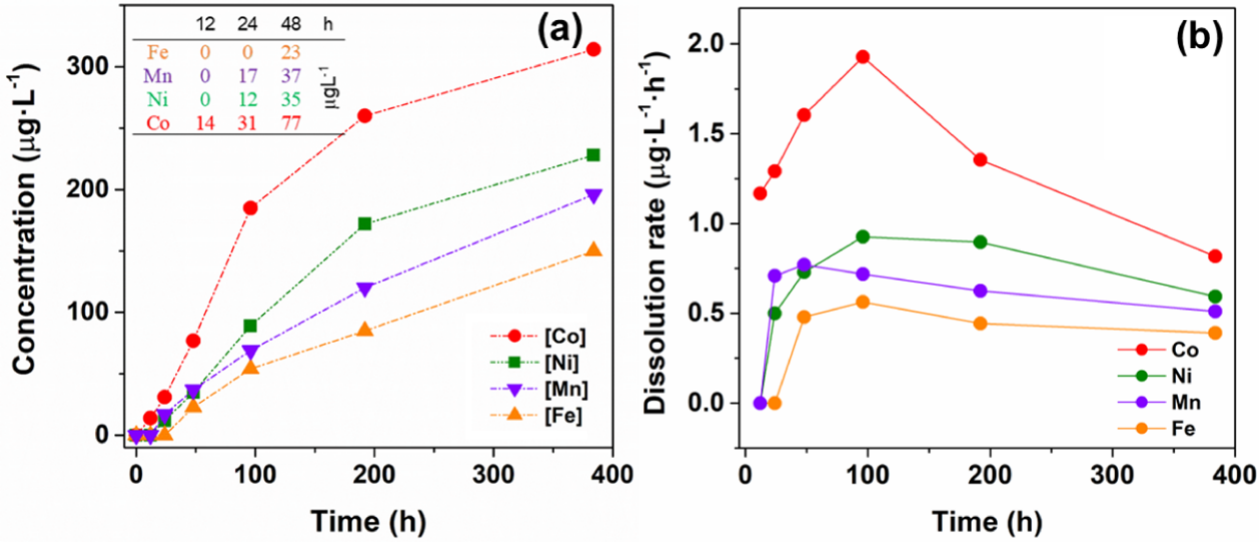
(a) Concentration of B cations in the supernatant 2M NaOH solution used for the stability tests and the corresponding (b) dissolution rate, both plotted as a function of the immersion time (the inset details the values for a shorter time).

The Pourbaix diagrams calculated for these materials, shown in Figure 2, provide a good explanation for these results. Both Co and Ni elements are likely to be present in solution at pH > 14 and 0 V vs. SHE, as ionized cobalt (HCoO_2_^–^) and nickel hydroxides (Ni­(OH)_4_^2–^), whereas the Mn and Fe lanthanum oxides should be stable under equivalent pH and overpotential conditions. In fact, the perovskite LF is the equilibrium phase at pH > 14 within a wide potential window. The La–Mn diagram indicates a stable phase of LM perovskites with a slight oxygen overstoichiometry, as expected for low temperature.^37^

**Figure 2:**
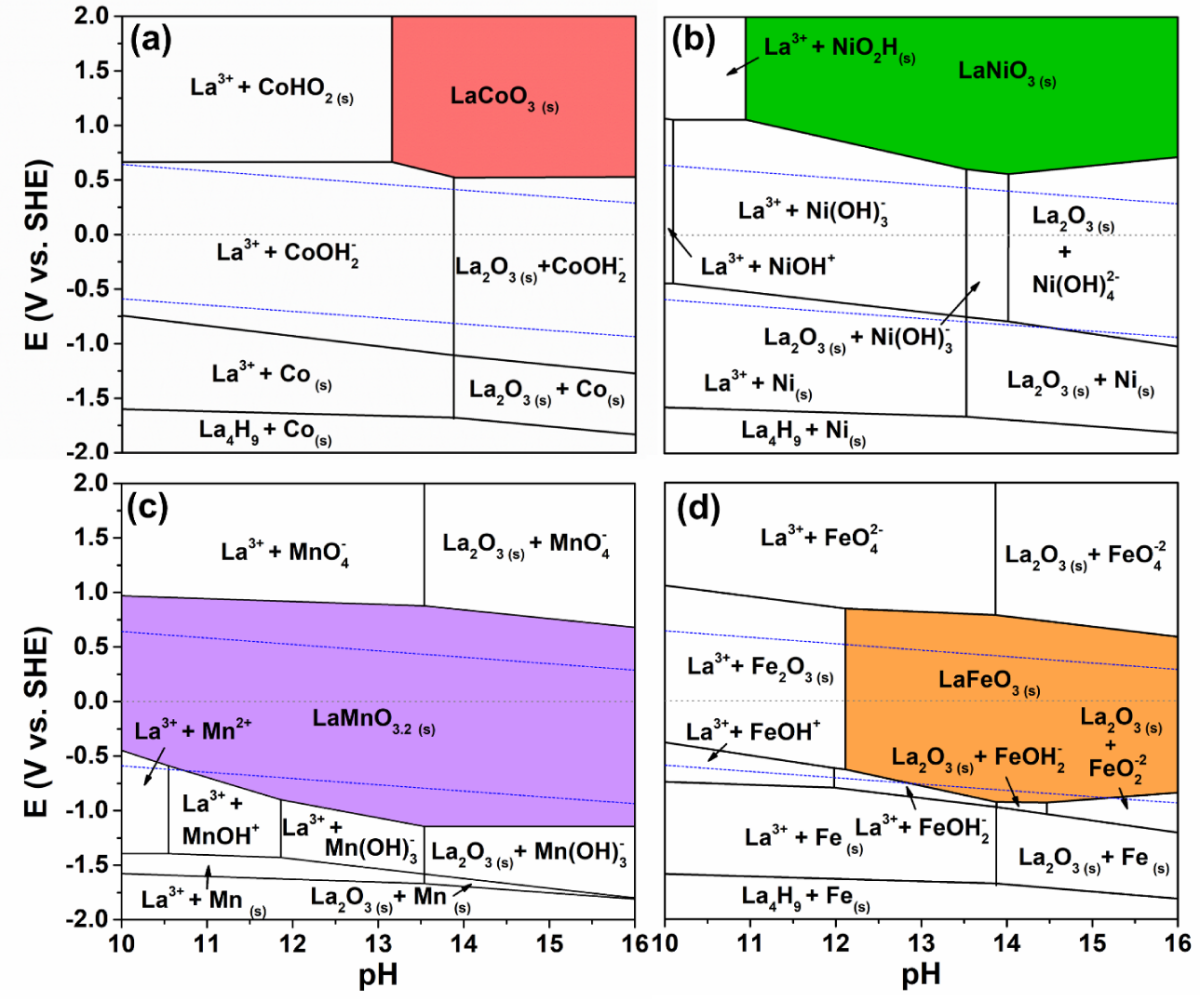
Pourbaix diagrams calculated at 25 °C with the concentration of La and transition metal offset to 10^–8^ mol·kg^–1^ for perovskites (a) LaCoO_3_, (b) LaNiO_3_, (c) LaMnO_3_, and (d) LaFeO_3_. The dashed lines correspond to water oxidation (top, 2H_2_O → O_2_ + 4H^+^ + 4e^–^) and reduction (bottom, 2H_2_O + 2e^–^ → H_2_ + 2OH^–^).

Since the transition metals leach into the alkaline solutions, one expects a lanthanum-rich phase to form somewhere. However, we could not detect any trace of La in the alkaline chemical attack solutions, and indeed the La–O Pourbaix diagram indicates La­(OH)_3_ (s) as the equilibrium phase at pH > 9 and over a wide potential window (at pH = 14, La is formed only below *ca*. −2.8 V vs SHE).^38^ As noticed in Figure S1, the XRD patterns did not reveal the presence of any secondary phase after the chemical attacks, and differences in the lattice parameters are minimal (Table S1). Therefore, lanthanum is probably forming a poorly crystallized (or even amorphous) film on the surface of the particles.

The Pourbaix diagrams shown in Figure 2 are calculated with a low lanthanum and transition metal concentration (10^–8^ mol·kg^–1^). Since the metal cations dissolve into the attacking solution, the progressive increase of the metal concentration with increasing reaction time is likely to affect the reaction equilibrium because the driving force for the reaction decreases due to the progressive increase of the transition metal concentration in the attack solution. The comparison of Figure 2 with the Pourbaix diagrams shown in Figure S2 illustrates the effect of the increase of the transition metal concentration from 10^–8^ mol·kg^–1^ to 10^–2^ mol·kg^–1^, with the latter depicting stability domains of the perovskite phases extended to both more cathodic and anodic potential. The effect is particularly noticeable in the cases of LaCoO_3_ and LaNiO_3_, in which the anodic boundaries are lowered to a less positive potential and intercept the stability domain of liquid water. The aforementioned decrease in the dissolution rate of the transition metal cations with increasing reaction time can thus be explained by changes in the thermodynamic equilibrium resulting from a variable metal concentration in the attack solution.

One may conclude that the equilibrium Pourbaix diagrams provide a sufficient explanation for the observed low alkaline stability of LC and LN. However, they fail to explain the apparent dissolution of LF and LM during longer reaction times. This could suggest that the surface composition of these materials is different from the nominal stoichiometric composition. In order to clarify the extent of the impact of the chemical attack on the bulk and surface properties of the materials, the pristine powders and those resulting from an alkaline attack for 384 h were submitted to systematic characterization by SEM, EDS, nitrogen sorption, and XPS.

SEM analysis reveals that there is no apparent impact of the alkaline treatment on the particle size, which remains basically unaltered in the ranges 250–300 nm for both LC and LN, and 100–150 nm for LM and LF (micrographs in Figure S3). This analysis further shows a certain level of agglomeration of the particles resulting from the high-temperature treatment. The particle coalescence is confirmed by the low values of the specific surface area estimated from nitrogen sorption isotherms (Table S2). If one takes the ratio between “particle size” and “specific surface area” as a crude indication of the level of particle agglomeration (the lower the ratio, the lower the agglomeration), one may conclude that the agglomerate microstructure of LM and LF is quite stable upon alkaline treatment, whereas the size of the LC and LN agglomerates tends to decrease. The reduction of the agglomerate size is likely to result from the alkaline dissolution of the material in the interparticle regions.

A TEM/EDS study was thus conducted to gain insight into the compositional differences between the surface and bulk of the pristine particles after exposure to the alkaline solution. Figure 3 shows these results highlighting the two regions of the particle analyzed by EDS: the center, where the signal is mostly coming from the bulk of the particle, and the particle edge and interparticle regions, where the signal should be originated mainly from the surface (typical spectra are shown in Figure S4). One notes that EDS in this context is a semiquantitative method (due to a variable interaction volume), and thus, the results are expressed in terms of the B:La atomic ratio (B is the transition metal in LaBO_3_) indicated for each of the corresponding particle regions.

**Figure 3:**
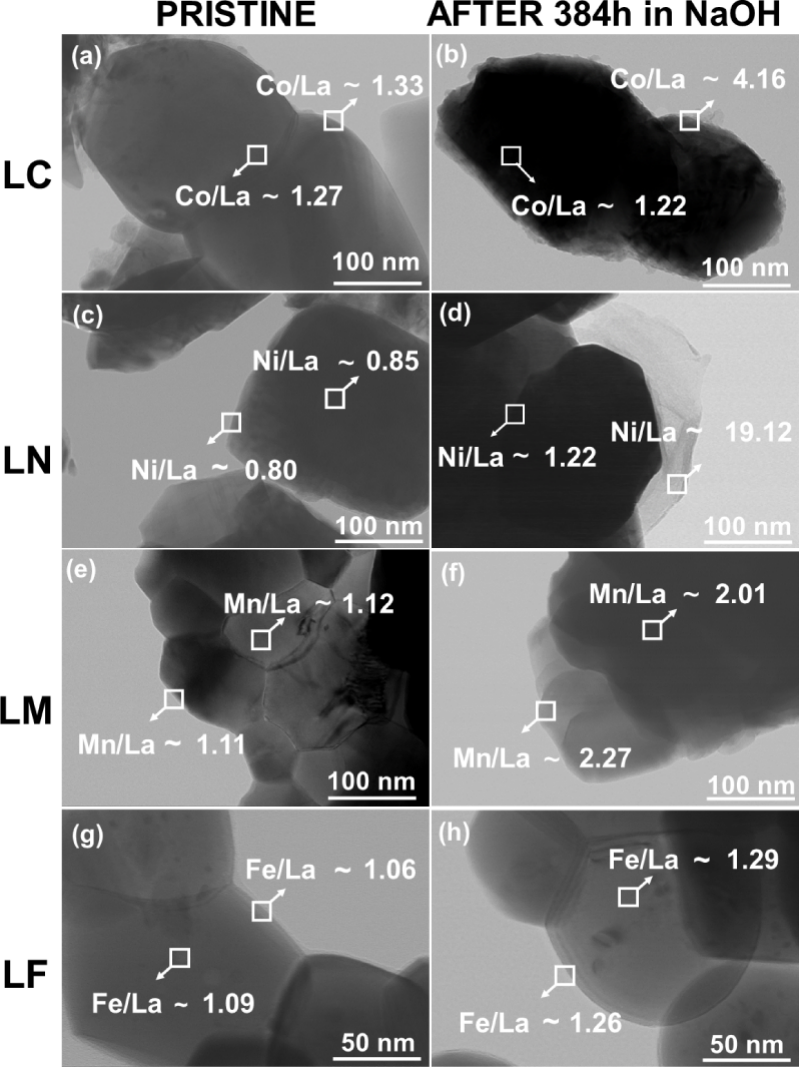
TEM micrographs for (a) LC-0, (b) LC-384, (c) LN-0, (d) LN-384, (e) LM-0, (f) LM-384, (g) LF-0, and (h) LF-384. The numbers correspond to the B:La atomic ratio determined by EDS in the squared regions (spectra in Figure S4).

In general, the results obtained for the pristine powders reveal a B/La ratio sufficiently close to unity for both the bulk and the surface. There are slight differences, but they do not have statistical significance. In turn, the analysis of the alkaline-treated samples suggests an increase in the B:La ratio with respect to the pristine materials. This increase is highly variable with composition and with the particle region, with the cobaltite and nickelate revealing, to a large measure, the greatest heterogeneity. Moreover, the analysis of the particle edges suggests an important enrichment of the surface with transition metal cations, noticeably upon a comparison of Figure 3a,c with Figure 3b,d. These results are consistent with the formation of amorphous hydroxides, such as HCoO_2_^–^ and Ni­(OH)_3_^–^ or Ni­(OH)_4_^2–^, as indicated by the Pourbaix diagrams depicted in Figure 2a,b. On the contrary, both the LM and LF show higher stability after treating with 2M NaOH, as no clear signs of Mn or Fe segregation to the surface could be identified by EDS. These results agree well with the XPS analysis discussed in the following section.

### Analysis of Surface Composition by XPS

XPS provides significant insight into the surface composition of perovskite samples, namely, the identification of the oxidation state of the transition metal and relevant information about various other chemical species, namely, lattice and adsorbed oxygen, hydroxyl groups, and adsorbed water. The whole XPS spectra of all samples show peaks corresponding to C 1s, O 1s, La 3d and 4p, and B cation 3s and 2p (Figure S5). Table 1 summarizes the results of the deconvolution analysis of the obtained XPS spectra as depicted in Figures S6–S8, on a comparison basis between as-prepared powders fresh (LB-0) and those treated in NaOH for 384 h (LB-384).

**Table 1:** Fraction of B Cations with O.E Oxidation State and Fraction of Oxygen Species, Both Obtained from XPS Spectra Collected Before and After Chemical Stability Tests in 2M NaOH Solution

	Oxidation state of B cation (%)			Oxygen species (%)			
Sample	O.E 2+	O.E 3+	O.E 4+	Lattice Oxygen	Adsorbed Oxygen	OH^–^ Species	Water Adsorbed
**LC-0**	27	73	-	37	16	38	9
**LC-384**	56	44	-	10	24	56	10
**LN-0**	18	82	-	28	17	39	16
**LN-384**	54	46	-	18	13	54	15
**LM-0**	-	92	8	55	23	12	10
**LM-384**	-	53	47	47	26	21	6
**LF-0**	32	44	24	52	15	19	14
**LF-384**	34	47	19	26	42	20	12

In all the samples, the La 3d spectra are characterized by a double peak for each spin–orbit component, attributed either to energy loss phenomena induced by intense O 2p–La 4f charge events or to the existence of mixing of electronic configurations, in agreement with the literature (Figure S6).^39,40^ However, the La 3d binding energy in LC-384 exhibits well-defined doublets, as shown by arrows in Figure S6a. This second peak implies the presence of La^3+^ cations in different environments, which are probably associated with La_2_O_3_/La­(OH)_3_.^17^ The Co 2p spectra in Figure S7a,b and the Ni 2p spectra in Figure S7c,d also reveal differences before and after the alkaline treatment. The fitting results provide a quantification of the relative fraction of the cobalt and nickel ions with oxidation states 2+ and 3+, as listed in Table 1. The most obvious explanation for this fact is the extensive coverage of the surface by ionized metal hydroxides such as HCoO_2_^–^ and Ni­(OH)_4_^2–^ after the alkaline treatment, as predicted by the Pourbaix diagrams and in line with the TEM/EDS observations of cobalt- and nickel-rich regions on the edges of the particles.

Contrary to the cases of LC and LN, where the average oxidation state of the cations is lower than 3+, the Mn 2p spectra indicate an average oxidation state higher than 3+ for the Mn cations. This occurs to charge balance the already-mentioned oxygen overstoichiometry (Figure S7e,f). Moreover, in the case of LM-384, one observes a substantial increase in the fraction of Mn^4+^ in comparison to LM-0, reaching nearly 50%. This translates to the increase of the average oxidation state of Mn from 3.08+ to around 3.47+, which corresponds to LaMnO_3.24_, in good agreement with the expected thermodynamically stable LaMnO_3.2_ phase obtained in the calculations for the Pourbaix diagram (Figure 2c).

While Co, Ni, and Mn seem to exist in two different oxidation states with variable fractions after exposure to the alkaline medium, the Fe 2p XPS spectra indicate the coexistence of Fe cations in three oxidation states (Table 1 and Figure S7g,h). In addition, the contribution of each Fe cation remains practically constant after immersion in the 2M NaOH solution for 384 h. This apparent structural stability is again in agreement with the stability foreseen by the Pourbaix diagram, although the surface composition may deviate from the nominal LaFeO_3_ perovskite stoichiometry.

The O 1s spectra signals for all samples are wide and asymmetric indicating that there are several kinds of oxygen chemical states: the lattice oxygen on the surface of the perovskite (O_latt_), the surface adsorbed oxygen species (O_ads_), hydroxyl species (OH^–^), and oxygen adsorbed in the form of molecular water (H_2_O) with different contributions after exposure to NaOH (see Table 1 and Figure S8).^41,42^ The LC and LN spectra reveal the presence of a substantial fraction of adsorbed hydroxyl species, which increase in the samples treated in alkaline conditions (LC-384 and LN-384) to account for more than 50% of the oxygen species, thus correlating well with the increase of the fraction of Co^2+^ and Ni^2+^ indicated by XPS and the formation of ionized hydroxides predicted by the Pourbaix diagrams (Figure 2a,b). Such an increase of OH^–^ species on the surface seems to occur paired with the decrease of lattice oxygen, also supporting the extended decomposition of LC and LN under alkaline conditions. The LM data show a similar trend for the OH^–^ species, but it is less pronounced than in LC and LN (Figure S8e,f). On the other hand, the lattice and adsorbed oxygen contribute to about 75% of the O 1s XPS signal in both LM-0 and LM-384. All in all, the results indicate a stable LM surface upon exposure under alkaline conditions, which tends to stabilize the thermodynamically expected LaMnO_3.2_ phase (La_5_Mn_5_O_16_). Regarding LF-0 and LF-384, one may ascribe the peaks at the highest binding energy to oxygen adsorbed via surface oxygen vacancies (Figure S8g,h).^37,39^ The area of both peaks increases for LF-384, which may indicate an increase in the surface oxygen vacancy concentration at high pH. The contributions at low binding energy remain basically unchanged between LF-0 and LF-384, indicating that any hydroxide species existing on the surface is little affected by pH, in good agreement with the Pourbaix diagrams and the greater chemical stability.

In summary, the XPS data show that the surface composition of the as-prepared LC-0 and LN-0 powders is considerably different from the nominal LaBO_3_ composition, showing a tendency to form hydroxylated species associated with the 2+ oxidation state of the transition metal. This configuration is not stable, and the high pH condition further decreases the average oxidation state observed in LC-384 and LN-384, which one can explain by assuming the formation of surface ionized metal hydroxides as intermediate species in the dissolution process observed in separate stability tests. The surface compositions of LM-0 and LF-0 also deviate from the nominal LaBO_3_ stoichiometry, but the effect of pH is much smaller with LM-384 and LF-384 showing relatively small differences, which is also in agreement with the Pourbaix diagrams and the stability tests.

### Electrochemical Measurements

In the face of the significant changes induced by the high pH on the phase stability, a linear scanning voltammetry study was carried out to assess the effect of the degradation of the material on the OER and ORR activity. Here, it is important to note that the experiments were conducted in clean electrolytes to minimize potential contamination of the measurement by the presence of dissolved transition metals, especially in the cases of LC and LN. Figure 4 compares the ORR and OER results obtained for the pristine LB-0 samples and for those exposed to the alkaline solution for 384 h, LB-384, while Table 2 summarizes the relevant parameters obtained from the LSV curves. Reference data for ORR (Pt/C) and OER (RuO_2_) electrodes as well as for the Vulcan mesoporous carbon support are also included.

**Figure 4:**
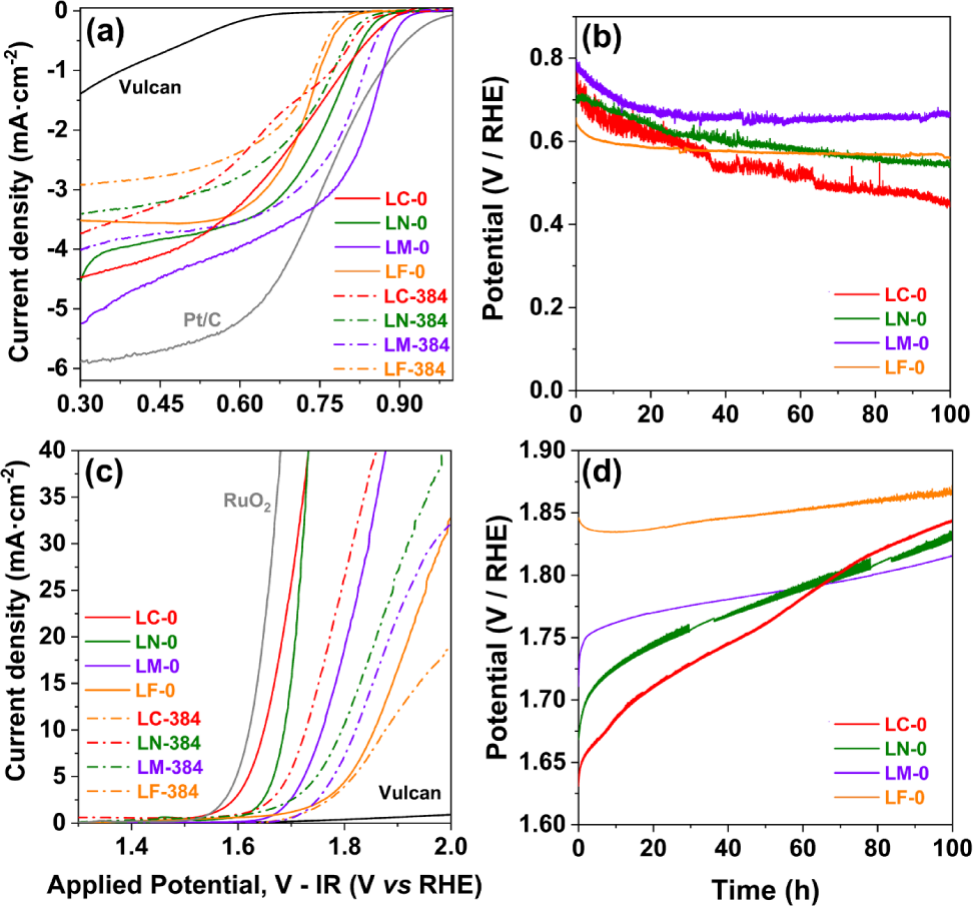
(a) Linear scanning voltammogram obtained at 1600 rpm comparing the ORR activity of the (solid lines) pristine and (dashed lines) perovskites treated in 2M NaOH solution for 384 h, (b) time dependent stability assessment under galvanostatic ORR conditions at *j* = −2.5 mA·cm^–2^, (c) linear scanning voltammogram obtained at 1600 rpm comparing the OER activity of the (solid lines) pristine and (dashed lines) perovskites treated in 2M NaOH solution for 384 h, and (d) time dependent stability assessment under galvanostatic ORR conditions at *j* = 10 mA·cm^–2^. Reference values for Vulcan carbon and conventional 20% platinum/carbon and RuO_2_ catalysts are included in the voltammograms.

**Table 2:** Comparison of ORR and OER Kinetic Parameters Obtained from Data in Figure 4 for the Various Electrodes in Pristine Condition (LB-0) and After 384 h Exposure to the 2M NaOH Alkaline Solution (LB-384)

	ORR			OER	
Sample	Onset potential (V vs RHE)	Tafel slope (mV·dec^–1^)	Number electrons (Koutechý–Levich)	Onset potential (V vs RHE)	Tafel slope (mV·dec^–1^)
**Pt/C**	0.98	95	3.4	-	-
**RuO**_**2**_	-	-		1.56	75
**LC-0**	0.89	45	3.8	1.58	97
**LC-384**	0.84	73	2.1	1.62	110
**LN-0**	0.83	54	3.8	1.63	69
**LN-384**	0.82	57	2.2	1.67	157
**LM-0**	0.91	45	3.8	1.67	69
**LM-384**	0.87	44	3.7	1.70	81
**LF-0**	0.79	52	4.0	1.72	164
**LF-384**	0.77	54	3.7	1.73	136

One first notes that the perovskite electrodes display significantly better ORR and OER performance than the Vulcan carbon support alone, although the latter displays ORR activity by itself. One also notes the underperformance of the perovskites when compared to the reference Pt/C material in ORR. This is to be expected given the latter are already optimized as commercial products, namely, on the specific surface area and its mixture with the carbon support. The fact that pristine LC shows comparable OER performance to RuO_2_, which is used here without addition of carbon, confirms the excellent activity of the cobaltite. The OER and ORR are analyzed separately in the following sections.

The voltammograms show that for low current densities (*j* < |−1.0| mA·cm^–2^) the ORR activity of the pristine LB-0 catalysts decreases according to LM ≫ LC > LN ≫ LF (solid lines in Figures 4a andS9a), which is in very good agreement with the volcano plot and the underlying e_g_ filling model proposed by Suntivich et al.^14^ According to these authors, the ORR is favored when the e_g_ orbitals of the transition metal are partially filled with (slightly less than) one electron. Thus, LF, with two e_g_ electrons, shows the lowest activity of the four materials, with relatively weak surface interactions between Fe and oxygen intermediates. In turn, LC-0 and LN-0 exhibit better ORR performance than LF-0, which is attributed to the predominance of B-site cations in the 3+ (73% and 82% for LC and LN, respectively, Table 1) and 2+ (27% and 18%) oxidation states, corresponding to slight overfilling above the single e_g_ electron. Moreover, LaCoO_3−δ_ and LaNiO_3−δ_ perovskites do not always permit the localization of the electron on the e_g_ orbital, either because of different symmetry between bulk (rhombohedral) and surface (tetragonal) in the case of LN or due to possible spin-state transition in the case of LC (Co^3+^ t^6^_2g_ e^0^_g_; t^5^_2g_ e^1^_g_; t^4^_2g_ e^2^_g_).^43^ LM-0 displays the highest ORR activity, again consistent with the presence of a single e_g_ electron in Mn^3+^. As shown in Table 1, 92% of the Mn cations in LM-0 are 3+, with the remaining 8% in the 4+ oxidation state. This would suggest that e_g_ orbitals are occupied by slightly fewer than one electron, which is the ideal configuration favoring ORR kinetics by further enhancing the O^2–^/OH^–^ exchange, in excellent agreement with the model proposed by Suntivich et al.^14^

Interestingly, the LSV for LC-0 quickly changes with increasing polarization, and for higher current density (*j* > |−2.0| mA·cm^–2^), one observes that the voltammogram approaches that of LF-0. The reason for this apparent degradation is not clear, but it is likely to be due to the poor chemical stability in alkaline media. In fact, all samples modified by exposure to the alkaline solution (LB-384, dashed lines in Figure 4a) display clearly worse ORR performance than the fresh LB-0 samples, with differences increasing with increasing polarization. All compositions are affected, and the relative ORR performance of LB-384 at *j* = |−1.0| mA·cm^–2^ can be described by the series LM > LN ≈ LC > LF. This change in relative positions reveals that the lower chemical stability at high pH (revealed by the alkaline stability tests and Pourbaix diagrams) correlates with a higher degradation of ORR activity. The LC and LN are the least stable samples, whereas the LF performance is only marginally affected by the 384 h alkaline treatment.

Such a trend is very well depicted by the galvanostatic stability tests of the LB-0 samples under ORR conditions (*j* = −2.5 mA·cm^–2^) shown in Figure 4b. Both LM-0 and LF-0 curves tend to stabilize after the initial decrease (about 20 h for LM-0, only 5 h for LF-0), whereas an approximately linear decrease of the potential with time is observed for LN-0 and LC-0 until the end of the measurements, after 100 h. The slope is distinctively greater for the cobaltite, which also displays the greater initial decrease, suggesting that major changes are occurring in the early stage of the measurements. It should be noticed that according to the calculated Pourbaix diagrams (Figure 2), the cathodic overvoltage observed during these measurements (roughly from −0.1 V vs. SHE to −0.33 V vs. SHE) should not be enough to promote the formation of metallic Co, Ni, or the solubilization of Mn or Fe hydroxyl anions, in agreement with the anodic galvanostatic plateau observed for LM-0 and LF-0 (Figure 4b). The galvanostatic curves agree with the noticed stronger degradation evidenced by the LSV of LC-0 (Figure 4a), and with the fact that only the cobaltite shows an increase in the Tafel slope (45 mV·dec^–1^ for LC-0 and 73 mV·dec^–1^ for LC-384, Table 2 and Tafel plots in Figure S9). In fact, except for LC-384, all samples have ORR Tafel slopes between 45 and 55 mV·dec^–1^, which fall within the lower range of those reported for this type of materials.^44^ Changes in the underlying ORR kinetics are also detected by the Koutecký–Levich analysis of the LSVs shown in Figure S10 and the corresponding linear fittings in Figure S11. This analysis indicates somewhat incomplete oxygen reduction in the case of the Pt/C reference electrode but is illustrative of the 4-electron charge transfer pathway for the four pristine LB-0 electrodes (Table 2). The alkaline treatment of the perovskites leads to a minor decrease of the number of electrons down to 3.7 indicative of the predominance of the 4-electron pathway, but to a major change in the case of the cobaltite and nickelate (2.1 and 2.2, respectively). The latter indicates a shift in the ORR charge transfer mechanism toward the 2-electron pathway, probably promoting the generation of hydrogen peroxide intermediates. In fact, the XPS data in Table 1 confirm that the Fe oxidation state in LF-384 remains nearly unchanged with respect to LF-0, whereas both LC-384 and LN-384 show a marked increase in the fraction of O.E 2+, becoming more metallic and indicating an overfilling of the e_g_ orbital, thus favoring peroxide formation.^14^ The case of the Manganite is interesting because the fraction of Mn^4+^ is higher in LM-384 (8% in LM-0 versus nearly 50% in LM-384), and this could further lower the filling of the e_g_ orbital, thereby limiting the OO^–^/OH exchange and decreasing the overall ORR kinetics.^14^ While the changes observed in the oxidation state of the surface B cations may indeed lead to the degradation of the ORR electrocatalytic properties of these lanthanum perovskites, particularly in the cases of LC and LN, the main reason for the poorer performance of the LB-384 samples in general should actually be associated with the decrease of the number of the surface transition metal cations actively participating in the ORR, and the concomitant formation of a lanthanum-rich layer on the surface, most likely the hydroxide.

A similar analysis leading to similar conclusions can be conducted on the OER data (Figure 4c and Table 2), including the interpretation based on the e_g_ partial filling model.^15^ In this case, however, if the number of e_g_ electrons is less than one, which implies maximum ORR performance, then the B–O^2–^ bond cannot be destabilized, resulting in a low regeneration of OH^–^ on the surface. Hence, the OER volcano plot displays a maximum in OER activity for slightly more than one electron in the e_g_ levels.^15^ This suggests that the presence of divalent cations in LC and LN may be beneficial for the OER, whereas the tetravalent manganese cations in LM result in a less favorable OER process. Furthermore, the two e_g_ electrons in LF have a less detrimental effect on OER compared to ORR. In good agreement with this prediction, the relative position of each composition on an OER performance scale derived from the voltammogram in Figure 4c (solid lines) varies according to LC > LN ≫ LM ≫ LF, with LC and LN showing distinctively better OER performance. In fact, the LC curve is actually very close to that of the reference material RuO_2_.

As observed for ORR, the samples exposed to the alkaline solution (dashed lines in Figure 4c) exhibit significantly higher OER potential when compared with pristine LB-0. The Tafel slopes (Table 2 and Figure S9c,d) are higher than for ORR, in agreement with the literature,^44^ and increase in the LB-384 samples with exception of LF (where it decreases), in line with the observed compositional trend for the increase of the anodic potential. The increase of the anodic potential and of the Tafel slope, seen as a measure of OER performance degradation of the LB-384 samples, is highest for the LN closely followed by LC and LM, with LF being the least affected material.

This compositional trend is fully reproduced by the OER galvanostatic stability tests of the pristine LB-0 samples. The results, presented in Figure 4d, depict a sharp increase of the anodic potential of LC, LN, and LM, respectively, during the first 15, 10, and 5 h and with decreasing magnitude in the series LC, LN, and LM in that series. LF-0 also shows a smaller initial step-change in potential during the first 2–3 h, but contrary to other materials, this change actually corresponds to an enhancement of OER performance. After this initial stage and until the end of the tests at 100 h, all samples depict a nearly linear increase of the potential with increasing time, which implies a reversal of the trend in the case of LF. The slope of the potential/time measures the OER degradation rate, which is highest for LC (1.8 mV·h^–1^), followed by LN (1.1 mV·h^–1^) and, at a much slower rate, LM (0.5 mV·h^–1^) and LF (0.3 mV·h^–1^). The compositional trend of these galvanic OER degradation rates is in remarkable agreement with the dissolution rates after 96 h, depicted in Figure 1b, establishing a strong correlation between both observations. This effect is once again attributed to compositional and morphological changes in surface composition, attributed to the displacement of transition metals, which diminishes the number of accessible active sites for the OER reaction.

## Conclusions

Powders of the LaBO_3_ transition metal perovskites (B = Co, Ni, Mn, Fe) were prepared by the well-established citrate complex method and exposed to strong alkaline solutions (2M NaOH) for up to 384 h to assess their chemical stability under high pH (greater than 14). The stability tests showed the preferential dissolution of Co and Ni, followed by Mn. LaFeO_3−δ_ shows the highest chemical stability but still reveals leaching of Fe cations after 100 h of testing. The SEM, TEM, and XPS results confirm that the underlying compositional changes are particularly important on the surface of the particles, where the transition metal cations tend to be depleted, thereby forming lanthanum-enriched regions. The calculated Pourbaix diagrams corroborate these results. Linear scanning voltammetry reveals a decrease of electrochemical activity for both ORR and OER of the four materials exposed to 2M NaOH solutions in comparison to the electrodes prepared with pristine powders, which is larger for the least stable cobaltite and nickelate. Time-dependent galvanostatic measurements carried out on the pristine samples for 100 h and without renewing the electrolyte fully confirm those observations. The overall electrochemical behavior of the tested compositional series can be understood on the basis of the number of electrons in the e_g_ orbitals before and after the alkaline exposure, according to the volcano plots and the underlying model behavior suggested by Suntivich et al.,^14,15^ where the highest ORR and OER activities correspond to the perovskite materials with slightly less than and slightly more than one electron in the e_g_ orbital. The degradation of the ORR and OER kinetic parameters is observed in the four materials decreasing in the series LC > LN > LM > LF, following closely the increasing chemical resistance to strong alkaline conditions. In this scenario, the ORR and the OER performance of LaFeO_3−δ_, initially the worse material owing to the two e_g_ electrons, tends to approach that of the better materials in the long term due to the much-enhanced chemical and redox stability under high pH conditions.

The full consequences of the material degradation, viz., the dissolution into the electrolyte solution, may be complex to assess as the interplay of numerous factors will eventually determine the electrochemical performance in the long term. For example, the changes in the surface composition leading to the formation of (oxy)­hydroxides as intermediate compounds of the dissolution process may improve the catalytic properties of the perovskite for some time. On the other hand, the dissolved transition metal cations may also change the electrolyte properties and thus the overall electrode performance,^45,46^ again hiding the true effects of the material dissolution in the long term. And finally, the dissolution of these types of materials in strong alkaline media is likely to be aggravated in compositions where the A cation is partly substituted by alkaline earths such as Sr,^25^ which is a typical approach to improve transport and catalytic properties (e.g., the state-of-the-art Ba_0.5_Sr_0.5_Co_0.8_Fe_0.2_O_3−δ_–BSCF).^26^ In fact, given the chemical similarity of Sr with Ca and Ba, these are also expected to easily leach into the electrolyte solution, which means that meaningful stability studies of such materials demand testing times well in excess of 100 h.

## Supplementary Material



## Notes

Experimental XRD patterns, SEM micrographs, EDS spectra, XPS spectra, Tafel plots, linear scan voltammograms and Koutechý–Levich plots for all the studied samples, Pourbaix diagrams calculated at 25 °C, space group and lattice parameters, and specific surface area tables (PDF)

## #

R. G.: Corporate R&D Centre, Bharat Petroleum Corporation Limited, Greater Noida 201306, India
