# Supplementary material for: Alkaline Stability of LaBO3 (B = Co, Ni, Mn, and Fe) Perovskites and Their Application as Bifunctional Oxygen Electrocatalysts for Electrochemical Devices
Source: J Phys Chem C Nanomater Interfaces. 2025 Aug 27;129(36):15998–6008. doi: 10.1021/acs.jpcc.5c03846 (PMC12821133; doi:10.1021/acs.jpcc.5c03846)
Supplement: Supplementary file 1 [file jp5c03846_si_001.pdf]

Supporting Information

Alkaline Stability of $LaBO_3$ (B = Co, Ni, Mn and Fe) Perovskites and Their Application as Bifunctional Oxygen Electrocatalysts for Electrochemical Devices

Rambabu Gutru[1,§], Daniel Muñoz-Gil[2,*], Mohamed Mamlouk[3], Filipe M. L. Figueiredo[1,*]

[1] CICECO-Aveiro Institute of Materials, Department of Physics, University of Aveiro, 3810-193 Aveiro, Portugal

[2] Departamento de Química Inorgánica I, Facultad de Ciencias Químicas, Universidad Complutense, 28040-Madrid, Spain

[3] School of Engineering, Newcastle University, Newcastle upon Tyne, United Kingdom, NE1 7RU

§ *Present address*: Corporate R&D Centre, Bharat Petroleum Corporation Limited, Greater Noida-201306, India

**Corresponding authors*: lebre@ua.pt (F.M.L.F.), dmunozgi@ucm.es (D.M.G.)

This Supporting Information contains eleven (11) Figures and two (2) Tables.

Figure S1. Experimental and calculated XRD patterns collected at room temperature for LC-0, LC-384, LN-0, LN-384, LM-0, LM-384, LF-0 and LF-384.

Figure S2. Pourbaix diagrams calculated at 25 °C for perovskites $LaCoO_3$, $LaNiO_3$, $LaMnO_3$ and $LaFeO_3$.

Figure S3. SEM micrographs for LC-0, LC-384, LN-0, LN-384, LM-0, LM-384, LF-0 and LF-384.

Figure S4. EDS spectra collected on the edge/surface of the particles depicted in the TEM micrographs of Figure 3 for fresh and alkaline attacked samples.

Figure S5. The whole XPS spectra for LC-0, LC-384, LN-0, LN-384, LM-0, LM-384, LF-0 and LF-384.

Figure S6. La 3d XPS spectra for LC-0, LC-384, LN-0, LN-384, LM-0, LM-384, LF-0 and LF-384.

Figure S7. Co 2p XPS spectra LC-0, LC-384, LN-0, LN-384, LM-0, LM-384, LF-0 and LF-384.

Figure S8. O 1s XPS spectra of LC-0, LC-384, LN-0, LN-384, LM-0, LM-384, LF-0 and LF-384.

Figure S9. Tafel plots comparing the ORR and OER activity of the LB-0 pristine perovskites and the LB-384 treated in 2 M NaOH solution for 384 h.

Figure S10. Linear scan voltammograms collected in oxygen-saturated conditions under variable electrode rotation rates for LB-0 and LB-384 samples.

Figure S11. Koutechý-Levich plots obtained from LSV in the ORR potential region for LB-0 and LB-384.

Table S1. Space group and lattice parameters by profile fittings of the XRD patterns.

Table S2. Specific surface area determined from nitrogen sorption isotherms of fresh perovskite powders and powders exposed to the 2M NaOH solution for 384 h.

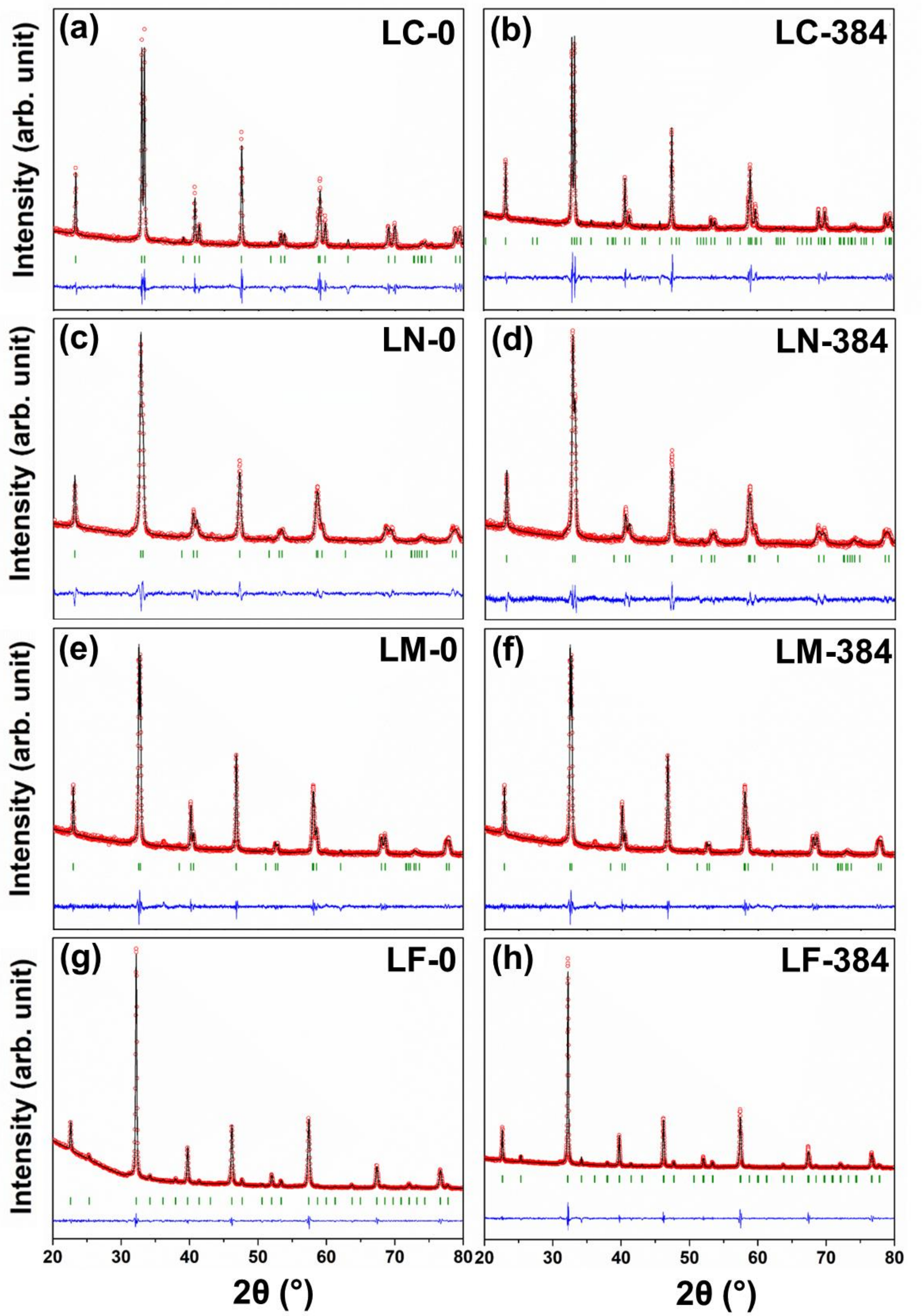

Figure S1. Experimental (red circles) and calculated (black continuous line) XRD patterns collected at room temperature for (a) LC-0, (b) LC-384, (c) LN-0, (d) LN-384, (e) LM-0, (f) LM-384, (g) LF-0 and (h) LF-384. Profile fittings carried out with the R-3c space group for Co, Ni and Mn perovskites and the R-3c space group for Fe perovskite. The vertical green bars indicate the positions of the Bragg peaks and the blue line at the bottom is the difference between the experimental and calculated patterns.

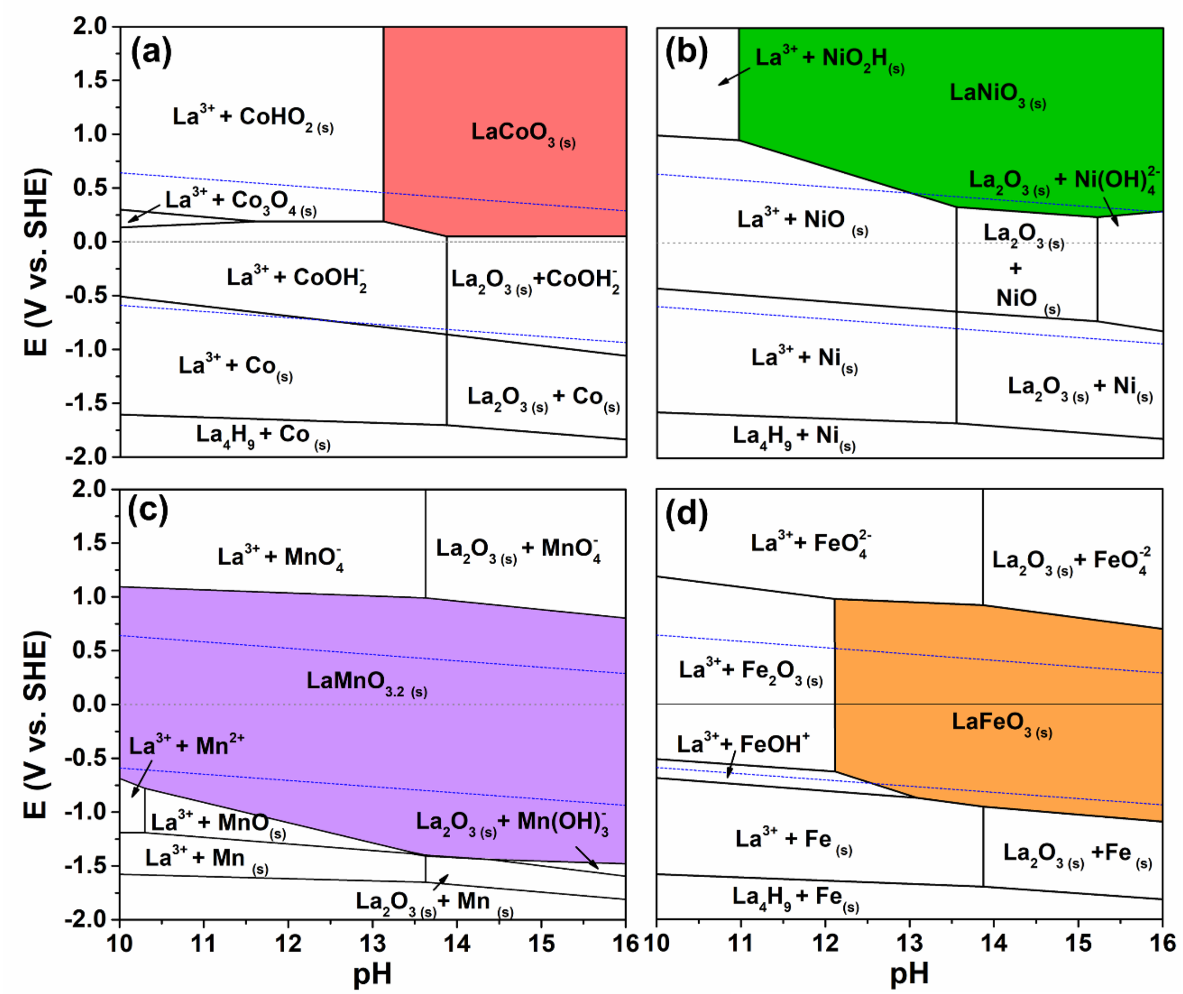

Figure S2. Pourbaix diagrams calculated at 25 °C with La concentration of 10^{-8} mol·kg^{-1} and metal concentration of 10^{-2} mol·kg^{-1} for perovskites a) $LaCoO_3$, b) $LaNiO_3$, c) $LaMnO_3$ and d) $LaFeO_3$. The dashed lines correspond to the water oxidation (top, $2H_2O \rightarrow O_2 + 4H^+ + 4e^-$) and reduction (bottom, $2H_2O + 2e^- \rightarrow H_2 + 2OH^-$). Note that the increase of the transition metal concentration from 10^{-8} mol·kg^{-1} (diagrams in Figure 2) to 10^{-1} mol·kg^{-1} (this Figure) in the solution enlarges the potential stability domain of all perovskites, but noticeably more in the cases of $LaCoO_3$ and $LaNiO_3$, which anodic boundary is here intercepting with the stability domain of liquid water, defined by the area between the two blue dashed lines.

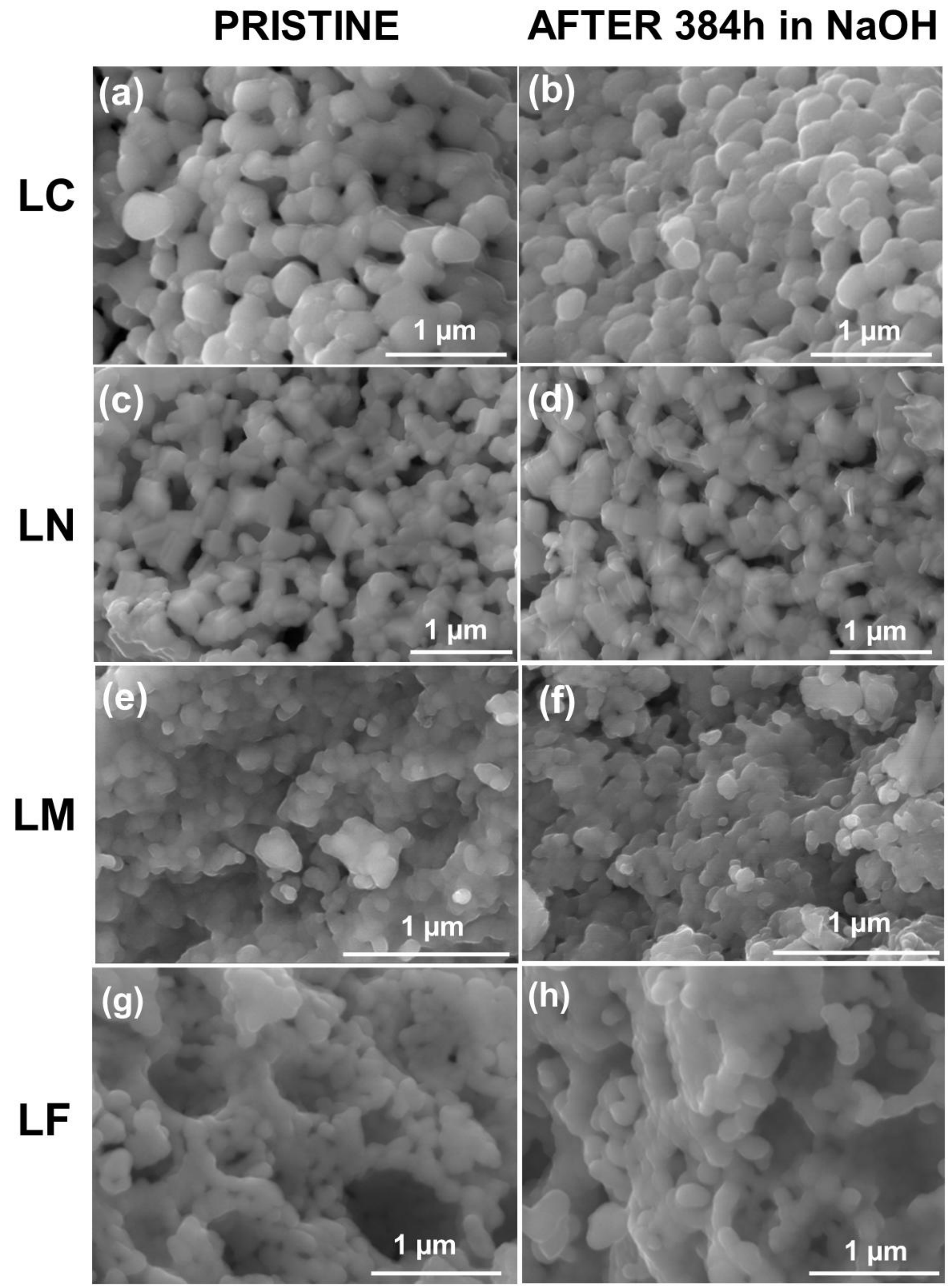

Figure S3. SEM micrographs for (a) LC-0, (b) LC-384, (c) LN-0, (d) LN-384, (e) LM-0, (f) LM-384, (g) LF-0 and (h) LF-384. Notice that the particle size remains unchanged upon alkaline treatment and is in the range [260-290] nm for LC, [260-300] nm for LN, [100-110] nm for LM, and [170-190] nm for LF.

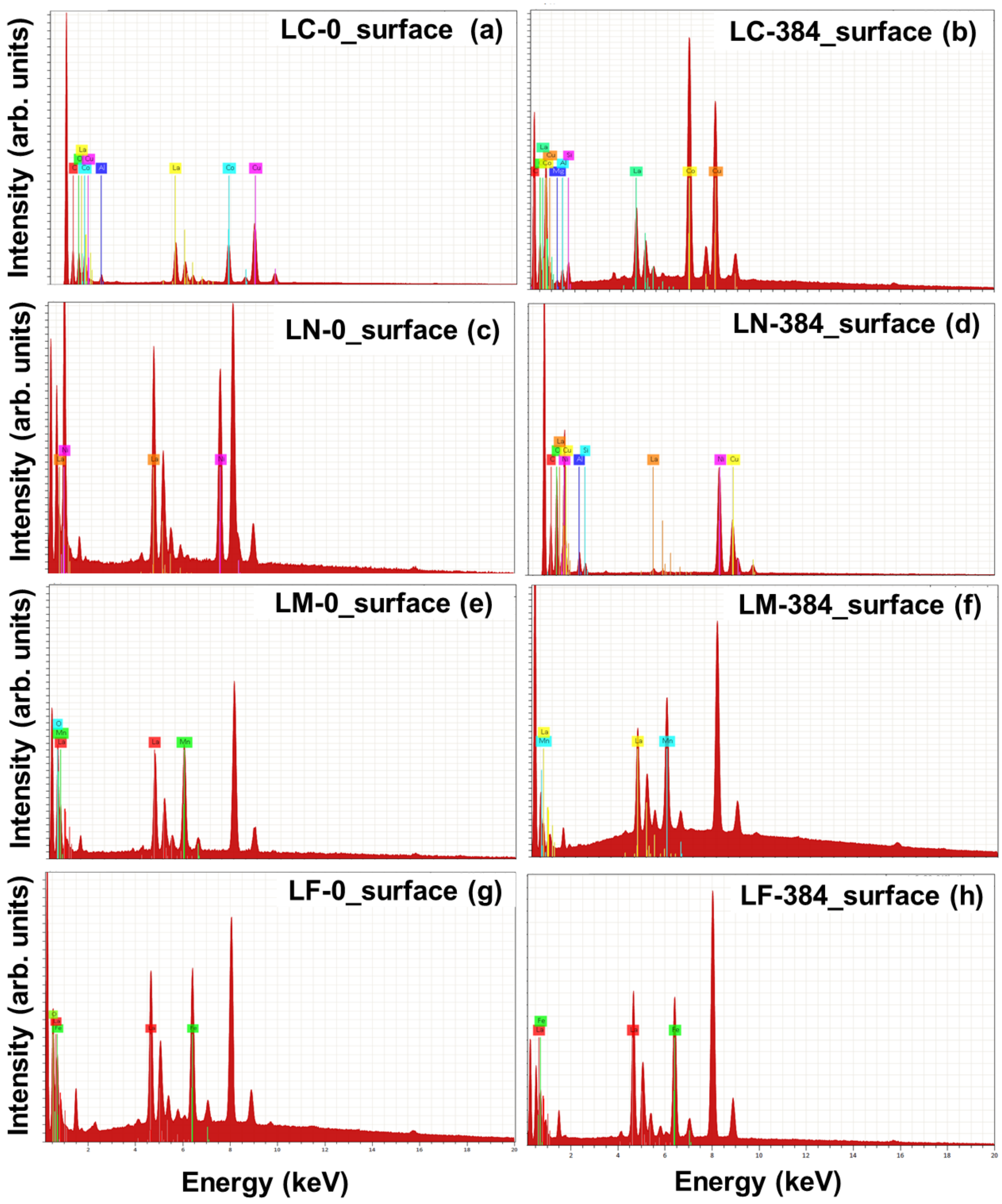

Figure S4. EDS spectra collected on the edge/surface of the particles depicted in the TEM micrographs of Figure 3 for (left) fresh and (right) alkaline attacked samples, identified as (a) LC-0, (b) LC-384, (c) LN-0, (d) LN-384, (e) LM-0, (f) LM-384, (g) LF-0 and (h) LF-384.

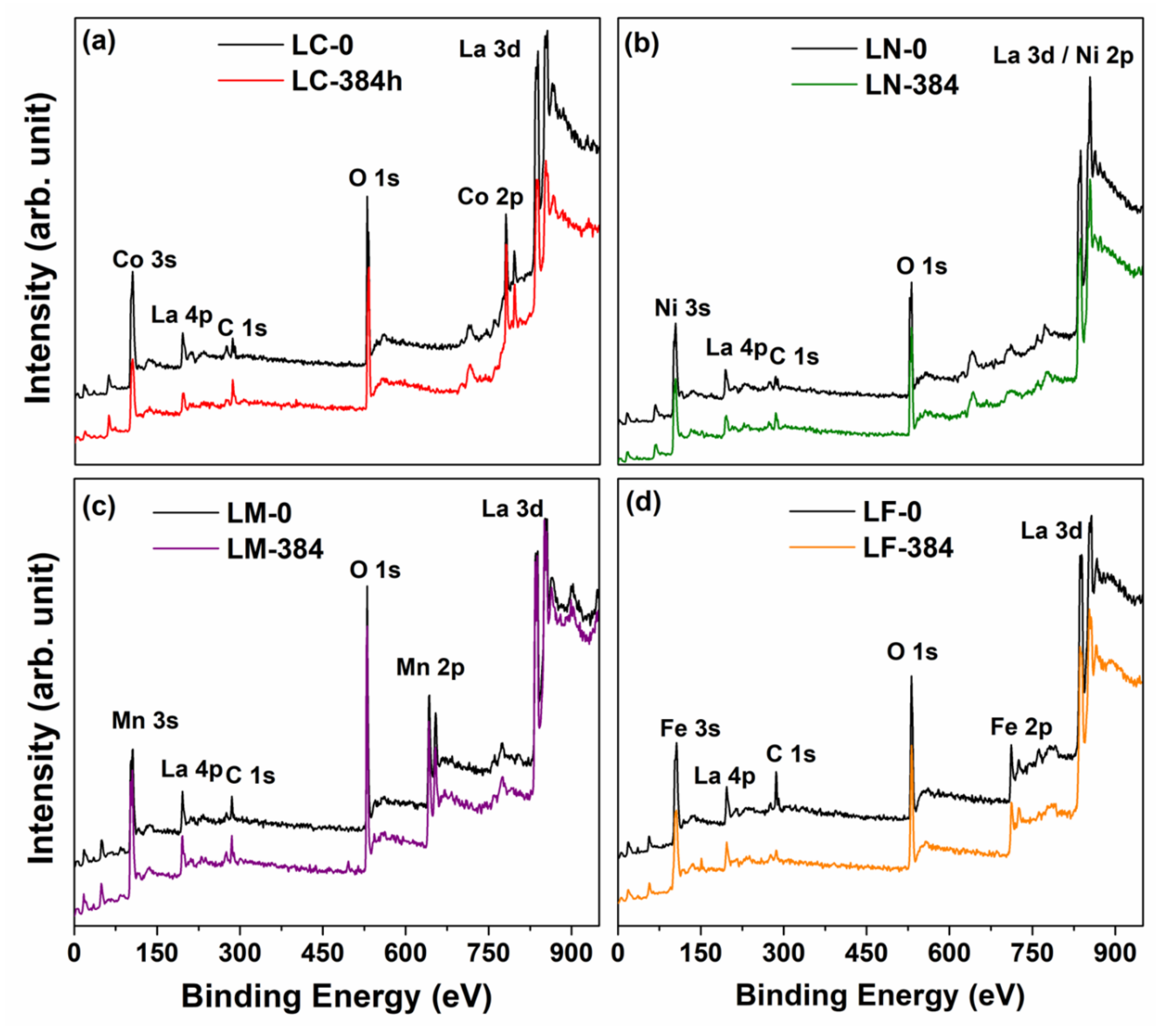

Figure S5. The whole XPS spectra of (a) LC-0 (black line) and LC-384 (red line), (b) LN-0 (black line) and LN-384 (green line), (c) LM-0 (black line) and LM-384 (purple line) and (d) LF-0 (black line) and LF-384 (orange line).

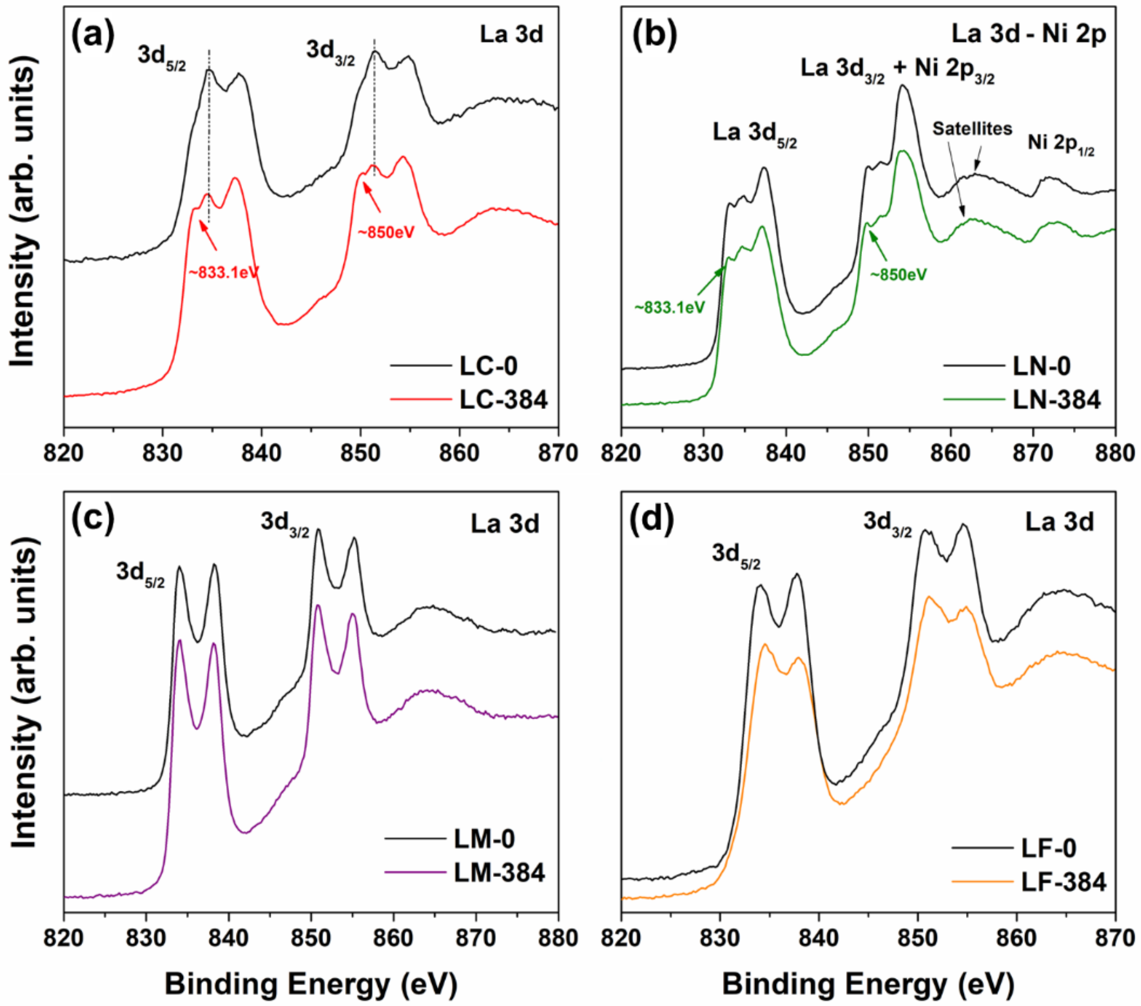

Figure S6. La 3d XPS spectra for (a) LC-0 (black line) and LC-384 (red line), (b) LN-0 (black line) and LN-384 (green line), (c) LM-0 (black line) and LM-384 (purple line), (d) LF-0 (black line) and LF-384 (orange line).

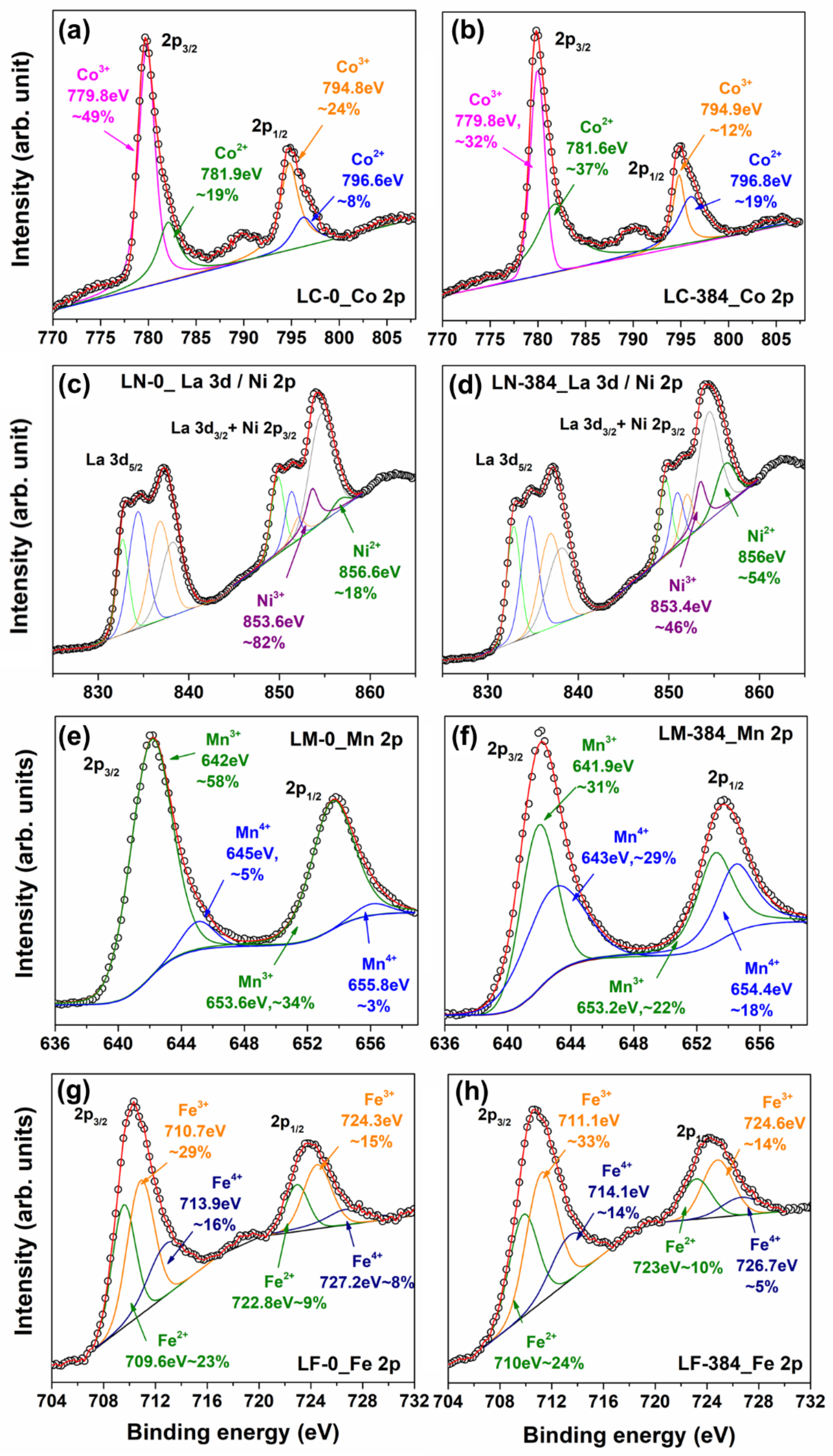

Figure S7. Co 2p XPS spectra of (a) LC-0 and (b) LC-384, Ni 2p XPS spectra of (c) LN-0 and (d) LN-384, Mn 2p XPS spectra of (e) LM-0 and (f) LM-384, and Fe 2p XPS spectra of (g) LM-0 and (h) LM-384. The coloured lines depict the deconvolution of the relevant species.

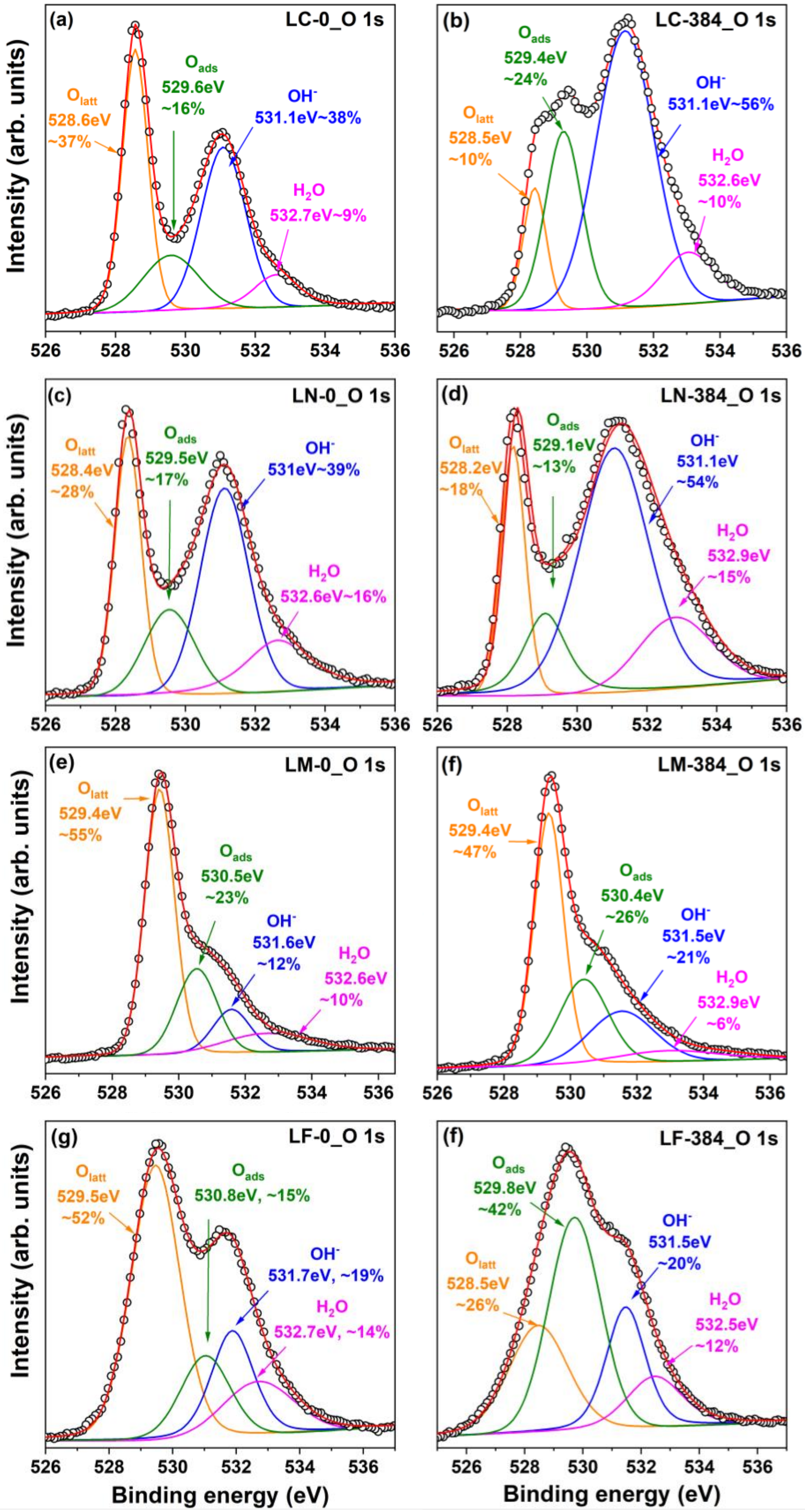

Figure S8. O 1s XPS spectra of (a) LC-0 and (b) LC-384, (c) LN-0 and (d) LN-384, (e) LM-0 and (f) LM-384, and (g) LF-0 and (h) LF-384, deconvoluted in the contribution of (orange) lattice and (green) adsorbed oxygen, (blue) hydroxyl groups and (magenta) adsorbed water.

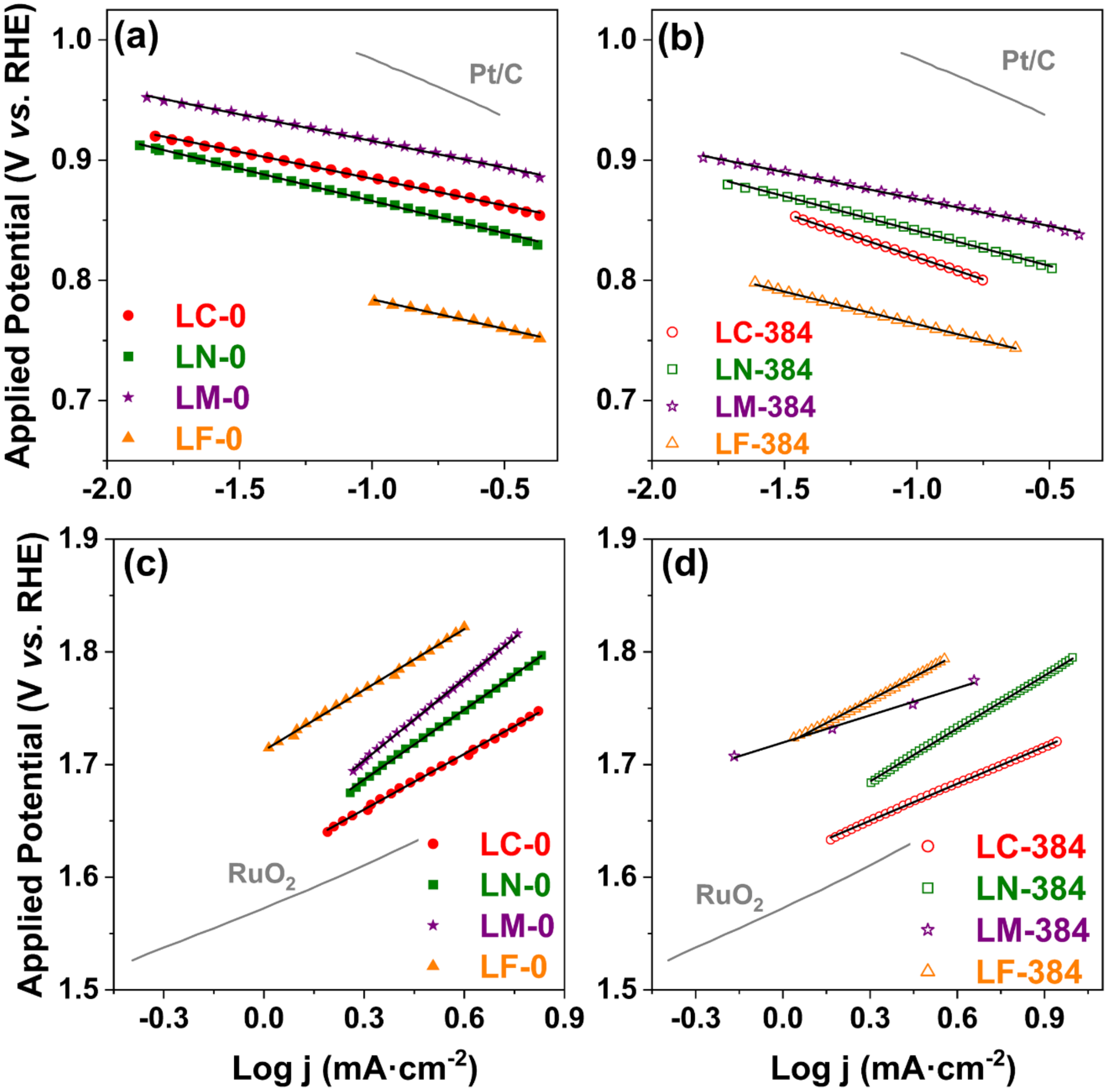

Figure S9. Tafel plots based in data from Figure 4 comparing the (A and B) ORR and (C and D) OER activity of the LB-0 pristine perovskites and the LB-384 treated in 2 M NaOH solution for 384 h. Data for conventional 20% Platinum/Carbon and RuO_2 fresh catalysts are also included as reference. The Tafel slopes are given in Table 2 of the manuscript.

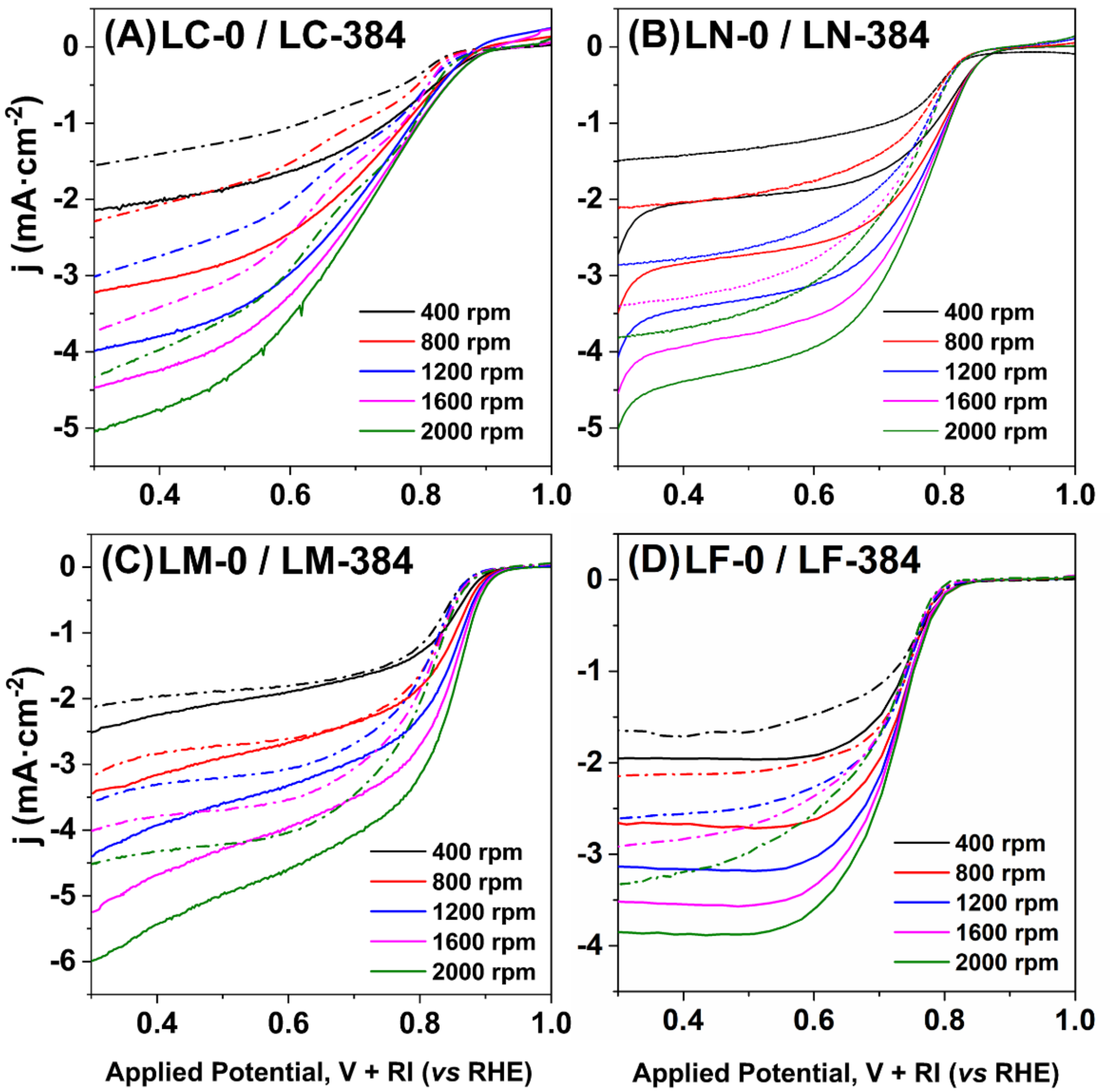

Figure S10. Linear scan voltammograms collected at 5 mV·s^{-1} in 0.5 M KOH and oxygen-saturated conditions under variable electrode rotation rates for (solid lines) pristine LB-0 and (dashed) alkaline-treated LB-384 samples.

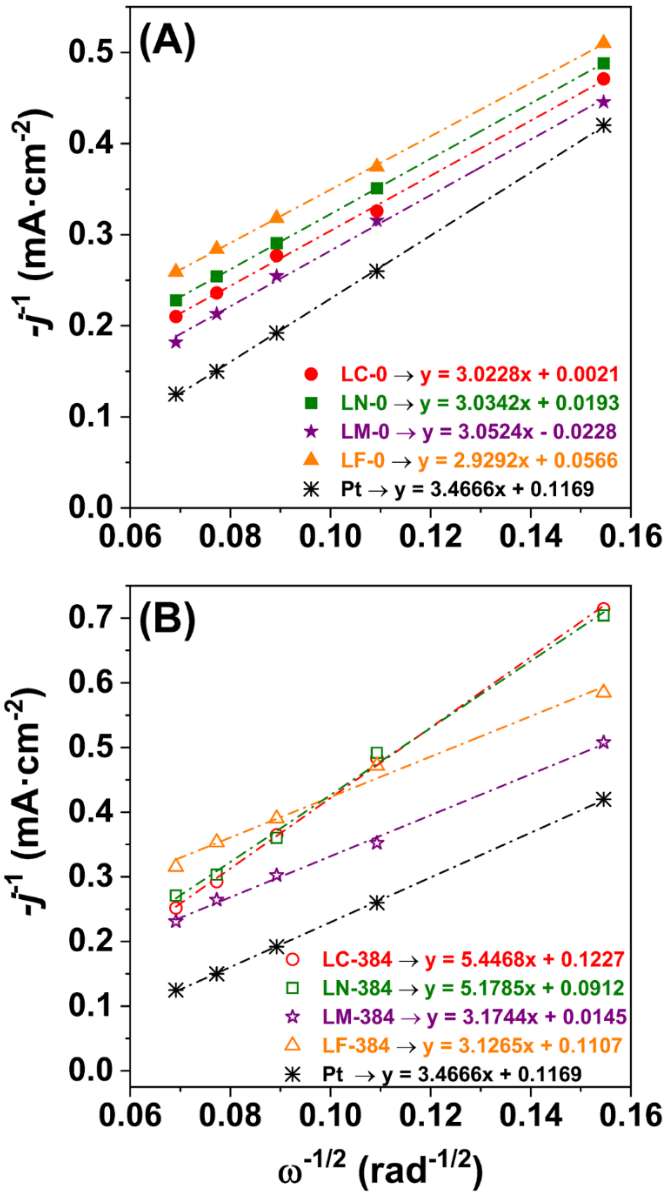

Figure S11. Koutechý-Levich plots obtained from LSV in the ORR potential region (0.4 V *vs*. RHE) for (A) the pristine LB-0 samples and (B) the LB-384 treated in 2 M NaOH for 384 h. The linear fitting parameters are given inside each figure and the estimated number of electrons involved in the ORR is provided in Table 3 of the manuscript.

Table S1. Space group and lattice parameters for each sample obtained by profile fittings of the XRD patterns.

Sample	Space group	Lattice Parameters(Å)		
		a	***b***	***c***
LC-0	Rhombohedral (R-3*c*)	5.4382 (2)	5.4382 (2)	13.0953 (7)
LC-384	Rhombohedral (R-3*c*)	5.4409 (2)	5.4409 (2)	13.1004 (5)
LN-0	Rhombohedral (R-3*c*)	5.4585 (5)	5.4585 (5)	13.1851 (9)
LN-384	Rhombohedral (R-3*c*)	5.4536 (2)	5.4536 (2)	13.1671 (7)
LM-0	Rhombohedral (R-3*c*)	5.5074 (2)	5.5074 (2)	13.3521 (6)
LM-384	Rhombohedral (R-3*c*)	5.5086 (3)	5.5086 (3)	13.3544 (7)
LF-0	Orthorhombic (P*bnm*)	5.5531 (2)	5.5556 (4)	7.8533 (2)
LF-384	Orthorhombic (P*bnm*)	5.5490 (2)	5.5581 (2)	7.8383 (5)

Table S2. Specific surface area (SSA) determined from nitrogen sorption isotherms of fresh perovskite powders and of the same powders exposed to the 2M NaOH solution for 384 h.

Sample	Specific Surface Area ($m^2 \cdot g^{-1}$)	
	**as-prepared (LB-0)**	**after 384h in 2M NaOH (LB-384)**
$LaCoO_3$	2.09	3.09
$LaNiO_3$	1.67	2.19
$LaMnO_3$	1.36	1.17
$LaFeO_3$	4.44	3.33
